# *Aspergillus flavus* Growth Inhibition and Aflatoxin B_1_ Decontamination by *Streptomyces* Isolates and Their Metabolites

**DOI:** 10.3390/toxins13050340

**Published:** 2021-05-08

**Authors:** Ixchel Campos-Avelar, Alexandre Colas de la Noue, Noël Durand, Guillaume Cazals, Véronique Martinez, Caroline Strub, Angélique Fontana, Sabine Schorr-Galindo

**Affiliations:** 1 UMR Qualisud, University of Montpellier, 34095 Montpellier, France; noel.durand@cirad.fr (N.D.); veronique.martinez@umontpellier.fr (V.M.); caroline.strub@umontpellier.fr (C.S.); angelique.fontana@umontpellier.fr (A.F.); sabine.galindo@umontpellier.fr (S.S.-G.); 2 CIRAD, UMR Qualisud, 34398 Montpellier, France; 3 IBMMUMR5247, University of Montpellier, CNRS, ENSCM, Place Eugène Bataillon, 34095 Montpellier, France; guillaume.cazals@umontpellier.fr

**Keywords:** actinobacteria, fungi, mycotoxins, enzymes, biodegradation, detoxification

## Abstract

Aflatoxin B_1_ is a potent carcinogen produced by *Aspergillus flavus*, mainly during grain storage. As pre-harvest methods are insufficient to avoid mycotoxin presence during storage, diverse curative techniques are being investigated for the inhibition of fungal growth and aflatoxin detoxification. *Streptomyces* spp. represent an alternative as they are a promising source of detoxifying enzymes. Fifty-nine *Streptomyces* isolates and a *Streptomyces griseoviridis* strain from the commercial product Mycostop^®^, evaluated against *Penicillium verrucosum* and ochratoxin A during previous work, were screened for their ability to inhibit *Aspergillus flavus* growth and decrease the aflatoxin amount. The activities of bacterial cells and cell-free extracts (CFEs) from liquid cultures were also evaluated. Fifty-eight isolates were able to inhibit fungal growth during dual culture assays, with a maximal reduction going down to 13% of the control. Aflatoxin-specific production was decreased by all isolates to at least 54% of the control. CFEs were less effective in decreasing fungal growth (down to 40% and 55% for unheated and heated CFEs, respectively) and aflatoxin-specific production, with a few CFEs causing an overproduction of mycotoxins. Nearly all *Streptomyces* isolates were able to degrade AFB_1_ when growing in solid and liquid media. A total degradation of AFB_1_ was achieved by Mycostop^®^ on solid medium, as well as an almost complete degradation by IX20 in liquid medium (6% of the control). CFE maximal degradation went down to 37% of the control for isolate IX09. The search for degradation by-products indicated the presence of a few unknown molecules. The evaluation of residual toxicity of the tested isolates by the SOS chromotest indicated a detoxification of at least 68% of AFB_1_’s genotoxicity.

## 1. Introduction

During food crop production, aside from the yield losses due to fungal infections, the appearance of mycotoxins represents a critical health risk for consumers, even more than the presence of pesticides and synthetic residues [1]. The term mycotoxin was first employed in the 1960s when investigating the cause of death of several turkeys in England after consuming a groundnut meal. The toxic molecule was later identified as aflatoxin B_1_ [2]. AFB_1_ is carcinogenic, mutagenic and immunosuppressive and is considered the most harmful mycotoxin [3,4,5,6]. *Aspergillus flavus* is the main producer of aflatoxin B_1_ (AFB_1_) and aflatoxin B_2_ (AFB_2_). As an opportunistic plant pathogen, it can develop on cereals such as wheat and maize, as well as on cotton, nuts and spices [7]. 

Aflatoxins constitute the second main hazard in France according to the Rapid Alert System for Food and Feeds (RASFF) with 34 alerts notified in 2018 [8]. Moreover, as climate change progresses, aflatoxin occurrence will increase [9]. Due to their potential health risk and their stability during industrial processing, the European Commission has established an AFB_1_ maximal content according to the type of food commodity. Limits for groundnuts before processing were set at 8 µg/Kg. For nuts, dried fruit, spices and maize before processing, the limit is 5 µg/Kg. Finally, for all the listed commodities after processing, plus all cereals and derived products, the limit decreases to 2 µg/Kg [10].

Prevention and curation techniques to avoid aflatoxin presence are being largely studied. Some examples of effective pre-harvest measures are the genetic modification of crops, crop rotation, irrigation, insect prevention and biocontrol by non-toxinogenic *Aspergillus*, such as Afla-guard^®^ and Aflasafe^®^, which are already commercialized. Post-harvest strategies include the precise control of storage conditions (low water activity and temperature), as well as sorting and eliminating contaminated grains [11]. However, as aflatoxin contamination persists, curative techniques must be implemented on foodstuffs, such as adsorption or physical and chemical reduction by degradation or inactivation. Unfortunately, these methods often lead to a decrease in nutritional properties, food quality reduction and increments in production costs. Several studies performed mainly on yeasts, lactic acid bacteria and actinobacteria showed that these microorganisms have the ability to decrease AFB_1_ [12,13]. However, the main reduction mechanism of AFB_1_ by lactic bacteria and yeasts is based on their ability to bind the mycotoxin to their cell walls, which makes this decontamination technique very limited [14,15]. Actinobacteria present an interesting natural and cost-effective alternative for the effective biodegradation of mycotoxins [1]. 

Actinobacteria are filamentous Gram-positive bacteria found in several ecological niches both on the soil and in water. *Streptomyces*, in particular, are largely researched for their ability to produce numerous molecules of interest, namely, antibiotics, antifungal compounds and enzymes, such as chitinases, which provide them with strong antagonistic capacities against fungal development [16]. The impact of actinobacteria on *Aspergillus flavus* growth has already been assessed, in addition to their ability to degrade AFB_1_ [14,17,18,19] and to inhibit its production. Indeed, some *Streptomyces* strains produce aflastatin A, blasticidin A and dioctatin A, three molecules that inhibit the aflatoxin biosynthetic pathway [20,21]. In addition, a *Streptomyces roseolus* strain was found to reduce AFB_1_ production by inhibiting aflatoxin gene cluster expression on *A. flavus* [22].

The major mechanisms involved in the degradation of AFB_1_ consist of a cleavage of the lactone group or a modification of the difuran ring or of the coumarin structure. Some enzymes known to be involved in AFB_1_ degradation are laccases, peroxidases, oxidases and reductases [14,23,24]. The main AFB_1_ degradation metabolites include aflatoxicol, aflatoxin B_2_a and aflatoxin D_1_, which are reportedly less toxic [15]. Moreover, two other modified forms, AFB_1_-8,9-epoxide and AFM_1_-8,9-epoxide, can bind to the DNA and provoke carcinogenic and mutagenic effects [25]. Hence, it is essential to ensure that AFB_1_ is effectively detoxified in less harmful molecules. For the evaluation of residual toxicity, *in vitro* tests allow evaluating AFB_1_ genotoxicity and mutagenicity, such as the Ames test and the SOS chromotest [26]. In the SOS chromotest, the bacterial gene *sfiA* expression, controlled by the SOS system for DNA damage, is monitored by assaying *β*-galactosidase activity [27]. It uses a mutant strain of *Escherichia coli* PQ37 which carries an *sfiA::lacZ* fusion and has a deletion of the normal *lac* region, meaning that β-galactosidase activity is strictly dependent on *sfiA* expression [28].

The aim of this work was to evaluate the *in vitro* interactions of 59 *Streptomyces* isolates, Mycostop^®^’s strain and their cell-free extracts (CFEs) with *Aspergillus flavus* and its aflatoxins. First, *Streptomyces* isolates were confronted with *A. flavus* to assess their direct impact on fungal growth and mycotoxin accumulation in the medium. Then, AFB_1_ degradation ability was studied during bacterial cell development in solid and liquid media, as well as by incubating the mycotoxins with the CFEs, to determine if degrading enzymes were produced extracellularly. Degradation by-products were researched by HPLC-MS, and their residual toxicity was evaluated by the SOS chromotest.

## 2. Results

### 2.1. Evaluation of the Antagonistic Activity of Streptomyces Isolates and Their Cell-Free Extracts (CFEs)

**Figure 1:**
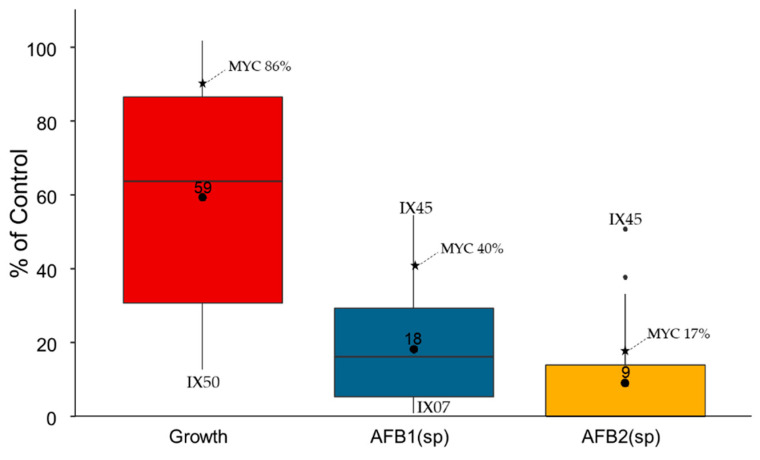
Effect of 59 *Streptomyces* isolates and Mycostop^®^ (MYC) on *Aspergillus flavus* growth and aflatoxin B_1_- and B_2_-specific production (accumulation) during dual culture on CYA medium at 25 °C for 8 days. The boxplot represents the distribution of the data expressed as a % of control (fungal growth and aflatoxin-specific production without bacteria).

The effect of fifty-nine *Streptomyces* isolates and Mycostop^®^’s *Streptomyces griseoviridis* strain (further referenced as MYC) on *A. flavus* growth and aflatoxin B_1_- and B_2_-specific production was evaluated by a dual culture assay (Figure 1). Fungal growth was decreased to 13% of the control (mean = 59%, median = 64%) by isolate IX50. AFB_1_-specific production (AFB_1_sp) was strongly reduced by all isolates (mean = 18%, median = 16% of the control), with at least 54% of the control for isolate IX45 and a negligible amount for isolate IX07 (0.7% of the control). The effect on AFB_2_sp was stronger and followed the same tendency as for AFB_1_sp, with half of the isolates provoking a high to full reduction in AFB_2_sp (mean = 9%, median = 0.08%). No isolate led to a specific overproduction of aflatoxins in the medium, meaning that an inhibition of AFB_1_sp occurred and/or that mycotoxins were degraded. Globally, most of the isolates had stronger antifungal and mycotoxin accumulation reduction properties than Mycostop^®^.

**Figure 2:**
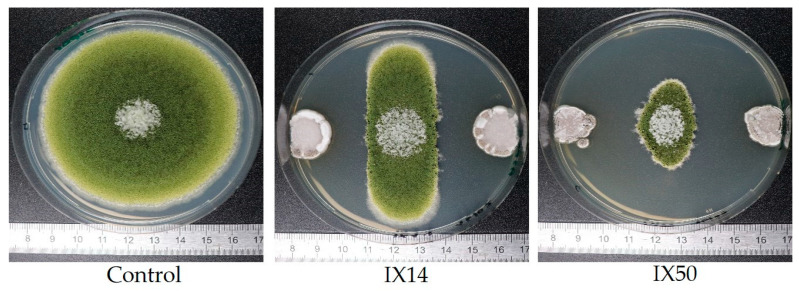
Growth profiles of *Aspergillus flavus* alone and during dual culture assay against *Streptomyces* isolates IX14 and IX50. Cultures were performed on CYA medium for 8 days at 25 °C.

Figure 2 shows the inhibition profiles of *A. flavus* growth by isolates IX14 and IX50 on CYA, where fungal growth was decreased to 37% and 13% of the control, respectively. As we can observe, the distance between bacterial and fungal colonies is almost the same in both cases; however, isolate IX50 strongly inhibited fungal growth on the edges, which highlights the importance of measuring the area of the whole fungal colony rather than the antagonistic distance. 

**Figure 3:**
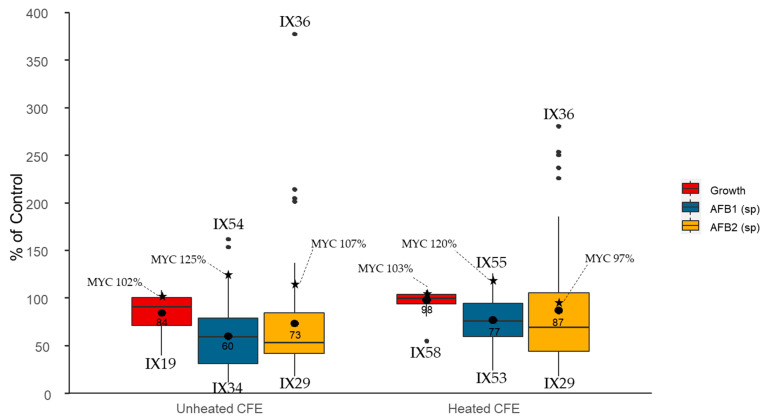
Effect of cell-free extracts (CFEs) of 59 *Streptomyces* isolates and Mycostop^®^ strain (MYC), added at 10% in the solid culture medium (CYA) of *Aspergillus flavus*, on fungal growth and aflatoxin-specific production (accumulation) after 8 days at 25 °C. The boxplot represents the distribution of the data expressed as a % of control (fungal growth and specific mycotoxin production without CFEs). Thermal treatment for heated CFEs: 100 °C for 10 min.

CFEs were added in the growth medium (10% *v*/*v*) of *Aspergillus flavus* with and without thermal treatment, in order to inactivate enzymes and discriminate between their potential impacts on fungal growth and AFB_1_sp. As shown by the boxplots in Figure 3, CFEs at 10% were less efficient at inhibiting fungal growth than bacteria in dual culture, with a maximal decrease to 40% of the control (mean = 84%, median = 91%) for unheated CFEs (IX19) and to 55% of the control (mean = 98%, median = 100%) for heated CFEs (IX58). 

Regarding the effect of CFEs, the results reveal a maximal decrease in AFB_1_sp for unheated CFEs with 11% of the control (mean = 60%, median = 59%) for IX34, and 23% (mean = 77%, median = 76%) for heated CFEs (IX53). Five unheated CFEs provoked an increase in AFB_1_sp, going up to 161% of the control (IX54). Heated CFEs increased AFB_1_sp in ten cases, going up to 125% of the control (IX55). CFEs were less effective at decreasing AFB_2_sp, with a mean of 73% (median = 53%) and 87% (median = 69%) of the control by unheated and heated CFEs, respectively. The maximal decrease in AFB_2_sp was observed for the CFE of IX29, with specific production lowered to 18% of the control, whether being heated or not. Ten CFEs caused a rise in AFB_2_sp, going up to 377% of the control for IX36, while 16 heated CFEs led to similar effects (up to 280% of the control). CFEs of Mycostop^®^ did not cause a significant change in fungal development nor in mycotoxin-specific production.

### 2.2. Mycotoxin Degradation Assay

**Figure 4:**
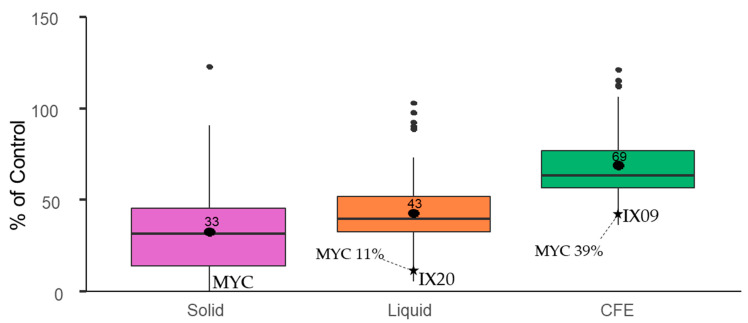
Degradation of aflatoxin B_1_ by *Streptomyces* isolates and Mycostop^®^ (MYC) cells in solid (CYA) and liquid (CYB) media after 10 and 5 days of culture, respectively, and by their unheated CFEs after 48 h at 25 °C and 180 rpm. The boxplot represents the distribution of the mycotoxin decrease as a % of control. Controls were included by adding AFB_1_ to CYA or CYB media, incubated under the same conditions.

*Streptomyces* isolates, MYC and their CFEs were evaluated regarding their capacity to degrade AFB_1_ at a concentration of 2 µg/mL. Boxplots in Figure 4 show that most isolates were able to degrade AFB_1_ on solid medium (mean = 33%, median = 32%) after 10 days of culture, down to undetectable residual amounts for MYC. In liquid medium, 5 days of culture allowed bacterial cells to degrade AFB_1_ down to 6% of the control for IX20 (mean = 43%, median = 39%). CFEs were less efficient at degrading the mycotoxin, with a maximal decrease down to 37% of the control for IX09 (mean = 69%, median = 64%). Mycostop^®^ also exhibited strong degradation capacities in liquid medium (11% of the control) and by its CFEs (39% of the control). 

### 2.3. Global Analysis of Strains: Cluster Analysis and Pearson Correlation Index 

In order to better visualize the specific characteristics and similarities of each of the 59 *Streptomyces* isolates and the Mycostop^®^ strain, the obtained results were interpreted using clustering analysis with a heatmap representation (Supplementary Materials Figure S1) and a correlational analysis by calculating Pearson’s correlation index for the two main clusters (Supplementary Materials Figure S2). The set of correlation coefficients (r) and their significance (*p*-value, *p*) are also presented in the Supplementary Materials (Table S1).

The heatmap in S1 shows the effect of each *Streptomyces* isolate regarding three types of interactions with *A. flavus* and its toxins:

- *flavus* growth in dual culture or with the addition of CFEs to the medium (Category 1);
- AFB_1_-specific production by *A. flavus* during growth in dual culture or with CFEs added to the medium (Category 2);
- AFB_1_ degradation by *Streptomyces* isolates in solid and liquid media, as well as by their CFEs (Category 3).

As the parameters analyzed had different magnitudes, a standardization was performed by transforming each percentage value to a Z-score, calculated by grouping the results of each of the three categories described before. A lower value of the Z-score represents a strong activity regarding the inhibition of fungal growth, of AFB_1_sp or its degradation. For the heatmap representation, a color scale was assigned to the Z-scores: clear yellow defines a strong activity (low Z-scores) and dark purple to black represents a lack of activity or an increase as compared to the control for AFB_1_sp (high Z-scores). Intermediate colors represent moderate activity. Finally, Euclidean distances were calculated considering the complete profile of the isolates within each category and sub-category which allowed clustering them according to their percentage of similarity. 

**Figure 5:**
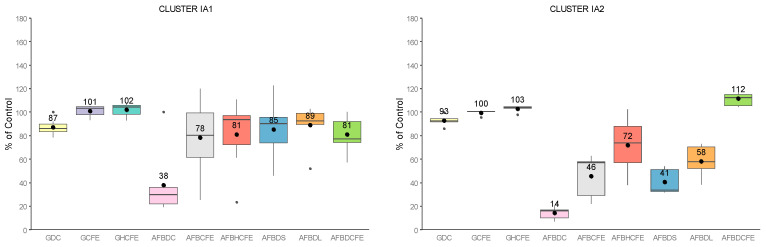

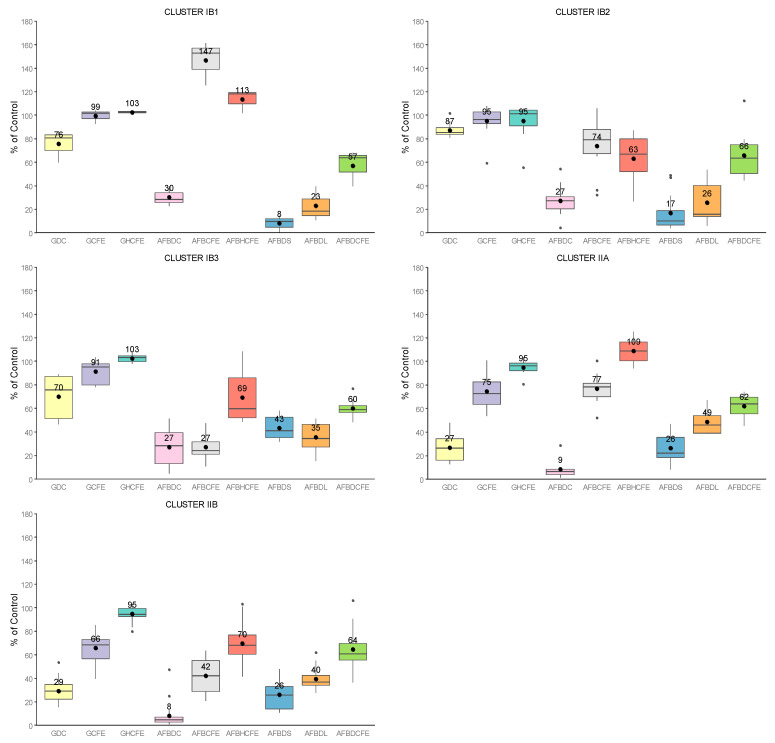
Boxplots of the effect of *Streptomyces* isolates and CFEs on *Aspergillus flavus* growth and AFB_1_-specific production (AFB_1_sp) (accumulation). Each sub-figure represents a subcluster of the heatmap from Figure S1. GDC = *A. flavus* growth in dual culture, GCFE = *A. flavus* growth vs. cell-free extracts (CFEs), GHCFE = *A. flavus* growth vs. heated CFEs, AFBDC = AFB_1_sp in dual culture, AFBCFE = AFB_1_sp vs. CFEs, AFBHCFE = AFB_1_sp vs. heated CFEs, AFBDS = AFB_1_ degradation by cells in solid medium, AFBDL = AFB_1_ degradation by cells in liquid medium, AFBDCFE = AFB_1_ degradation by CFEs.

*Streptomyces* isolates were separated into two main clusters and seven subclusters according to their effects recorded in each assay. Their principal features were evidenced by plotting the distribution of the measured parameters for each subcluster (Figure 5), and their main characteristics are summarized in Table 1. 

The following description of subclusters is based on the average of each evaluated category. Fungal growth inhibition during dual cultures (GDC) was mainly achieved by isolates from cluster II, which were able to decrease fungal development down to 27% of the control (IIA), with the most effective isolates being IX50 and IX23. Isolates from cluster I had a more limited effect, except for IB3 which decreased it down to 70% of the control and IB1 with 76% of the control. CFEs’ antifungal activity (GCFE) followed the same tendency as the dual culture assay, with efficiency mostly limited to cluster II, since fungal inhibition went from 91% for cluster I (IB3) to 68% of the control for cluster II (IIB). Isolates IX19 and IX18 (subcluster IIB) were the most effective CFEs at decreasing fungal growth. The thermal treatment (GHCFE) ruled out any antifungal activity for all CFEs. 

Regarding AFB_1_sp, all subclusters strongly affected toxin accumulation when confronted in the dual culture assay (AFBDC), with reductions ranging from 38% (IA1) to 8% (IIB) of the control on average. Isolates IX07 and IX31 (subcluster IIB) had the strongest mycotoxin reduction activity, with an almost complete decrease in AFB_1_sp (0.71% and 0.83% of the control, respectively). The impact of CFEs on AFB_1_sp (AFBCFE) was less pronounced, and variable effects could be noted: from a strong reduction for subcluster IB3 (27%)—particularly isolates IX34 and IX06—to an intermediate reduction for IA2 (46%) and IIB (42%), and a limited reduction for subclusters IIA (77%), IB2 (74%) and IA1 (78%). The subcluster IB1 exhibited singular properties with a strong enhancement of AFB_1_sp, going up to 141% of the control on average. The thermal treatment (AFBHCFE) generally decreased the influence of CFEs on AFB_1_sp in all subclusters, except for IB2 (74% vs. 63% of the control after thermal treatment).

AFB_1_ degradation was a common trait among all subclusters under both culture conditions, but it was usually higher in solid medium (AFBDS) rather than in liquid medium (AFBDL), except for subcluster IB3. Subcluster IA1 had the weakest degradation abilities in solid medium (85% of the control), whereas IB1 and IB2 exhibited the strongest, with 8% and 17% of the control, respectively. Other subclusters ranged within 26% (IIA and IIB) to 43% of the control (IB3). The strongest degradation was caused by the MYC strain (IB1) and by isolates IX28, IX20 and IX03 (IB2). Finally, CFEs’ degradation abilities (AFBDCFE) were usually lower than the degradation by the cells and comparable between the subclusters, ranging within 57 to 66% of the control, except for subcluster IA1 (81% of the control), where its degradation was equivalent to the degradation by the cells. The most effective CFEs regarding AFB_1_ degradation included those produced by IX09 (IIB), MYC (IB1) and IX45 (IB2). Subcluster IA2 was peculiar as it was the only one having substantial degradation properties by the cells (41% in solid medium and 58% in liquid medium) but no effect in the CFEs. 

The Pearson correlation analysis shown in Figure S2 (Supplementary Materials) indicates a positive correlation coefficient in cluster I between the effect of unheated and heated CFEs on AFB_1_sp (*r* = 0.6, *p* < 0.001), as well as between bacterial cells’ ability to degrade pure AFB_1_ in solid and liquid media (*r* = 0.8, *p* < 0.001). A moderate positive correlation is presented between the ability of *Streptomyces* isolates to degrade pure AFB_1_ in liquid medium and the degrading capacity of their CFEs (*r* = 0.5, *p* = 0.002). Cluster II exhibits a strong positive correlation between unheated and heated CFEs regarding their impact on *A. flavus* growth (*r* = 0.6, *p* < 0.001) and on AFB_1_sp (*r* = 0.8, *p* < 0.001). A moderate positive correlation also exists between the inhibition of fungal growth in dual culture and AFB_1_ degradation by bacterial cells in solid (*r* = 0.6, *p* = 0.0012) and liquid media (*r* = 0.4, *p* = 0.03). A slight positive correlation appears in dual culture between the inhibition of *A. flavus* growth and the reduction in AFB_1_sp (*r* = 0.5, *p* = 0.02). The dendrograms presented at the bottom of Figure S2 illustrate the relationship and proximity between the results of the *Streptomyces* isolates and their CFEs for the different parameters evaluated. Parameters in cluster I are slightly more closely related (~18% maximum Euclidean distance) than those in cluster II (~21% maximum Euclidean distance). In both clusters, parameters are associated within three main groups, but associations vary between them. AFB_1_ degradation by bacterial cells in solid and liquid media is close in cluster I, with a distance of 4%, while in cluster II, they are separated by 21%. In cluster II, the effect of *Streptomyces* isolates on AFB_1_-specific production in dual culture is closely related to the degradation in solid medium by bacterial cells (3%).

### 2.4. Search for Degradation By-Products

**Figure 6:**
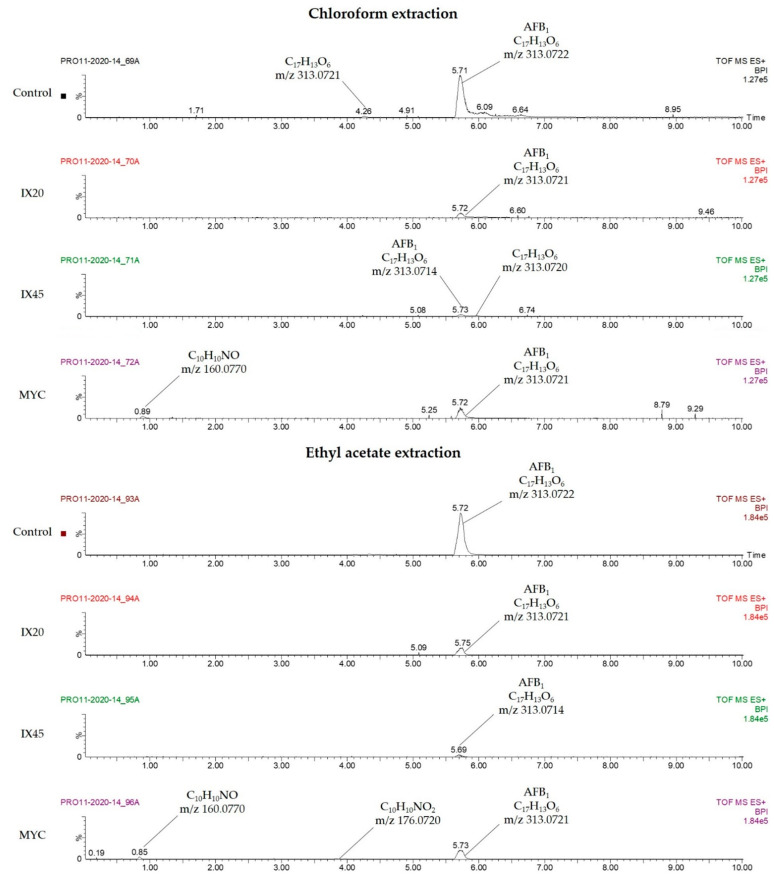
Mass spectrometry spectral data for aflatoxin B_1_ remaining after 12 days of incubation at 25 °C and 180 rpm with *Streptomyces* isolates IX20 and IX45 and MYC strain in CYB medium. Controls consisted of CYB with AFB_1_, incubated without bacteria, under the same conditions. Spectra were obtained for the extraction with two solvents: chloroform (**top**) and ethyl acetate (**bottom**). The peaks of the medium for the control and samples without AFB_1_ were subtracted as blanks.

Two isolates (IX20, IX45) and the MYC strain, with strong degrading capacities in liquid culture (with a decrease down to 6%, 10% and 11% of the control, respectively), were selected for the search of degradation by-products of AFB_1_ (8 µg/mL). The main breakdown products known to be issued from microbial degradation such as aflatoxicol, aflatoxin B_2_a and aflatoxin D_1_ and D_2_ were searched by HPLC-MS. Figure 6 presents the resulting spectra of the mass spectrometry analysis with the peaks and molecular formulas corresponding to the found unknown compounds, as well as their *m/z*.

Spectral data were obtained by extracting the supernatant with chloroform or ethyl acetate. Peaks were normalized in order to distinguish the decrease in AFB_1_ by the *Streptomyces* isolates, and to approximatively compare the amount of the detected molecules. In both extractions, a clear decrease in AFB_1_ peaks is observed for the bacterial cultures, in comparison to the control.

Chloroform extraction spectra exhibit ion peaks at *m/z* 313.0722 and 313.0721 for the control, corresponding to AFB_1_ and to what seems to be an AFB_1_ isomer, respectively. For IX20, only the ion peak of AFB_1_ at m/z 313.0722 is observed. For IX45, ion peaks at *m/z* 313.0714 and 313.0720 are present, corresponding to AFB_1_ and an AFB_1_ isomer, respectively. For MYC, ion peaks at *m/z* 313.0721 and 160.0770 are present, corresponding to AFB_1_ and to an unknown molecule of formula C_10_H_10_NO, respectively.

Ethyl acetate extraction spectra show an ion peak at *m/z* 313.0722 for the control, corresponding to AFB_1_. For IX20 and IX45, only the ion peak of AFB_1_ at *m/z* 313.0721 and 313.0714, respectively, is present. For MYC, ion peaks at *m/z* 313.0721, 176.0720 and 160.0770 are present, corresponding to AFB_1_, to a first unknown molecule of formula C_10_H_10_NO_2_ and to a second unknown molecule of formula C_10_H_10_NO, which was also observed for the MYC culture extracted with chloroform.

### 2.5. Evaluation of Residual Toxicity

To ensure the effective detoxification of AFB_1_ by the tested *Streptomyces* isolates and MYC, the liquid cultures resulting from the degradation by-products assay were used to verify the absence of residual toxicity, using the SOS chromotest method. The control was incubated under the same conditions as the samples, and standard AFB_1_ was prepared the same day as the assay. Table 2 presents the residual AFB_1_ content of the degraded samples and the SOS induction factor (IF) values from the SOS chromotest assay, as well as their corresponding percentages. IF values of *Streptomyces* cultures indicated that they were able to decrease the genotoxicity of 8 µg/mL of AFB1 (IF = 7.13) to 32% of the control (IF = 2.26) for IX20, 28% (IF = 1.99) for IX45 and 31% (IF = 2.18) for MYC. Thus, IX45 caused the strongest decrease in genotoxicity. A common interpretation of IF values indicates that a value lower than 1.5 is not genotoxic. In this study, degradation IF values were superior to 1.5. However, a decrease of about a third in genotoxicity is validated in comparison to the control. The concentration of AFB_1_ of the control, incubated under the same conditions as the samples, and its corresponding IF decreased slightly (~15%) during the incubation period, as compared to the AFB_1_ standard. Thus, about 15% of the observed genotoxicity decrease is due to an inherent degradation of AFB_1_ during the incubation time. 

**Figure 7:**
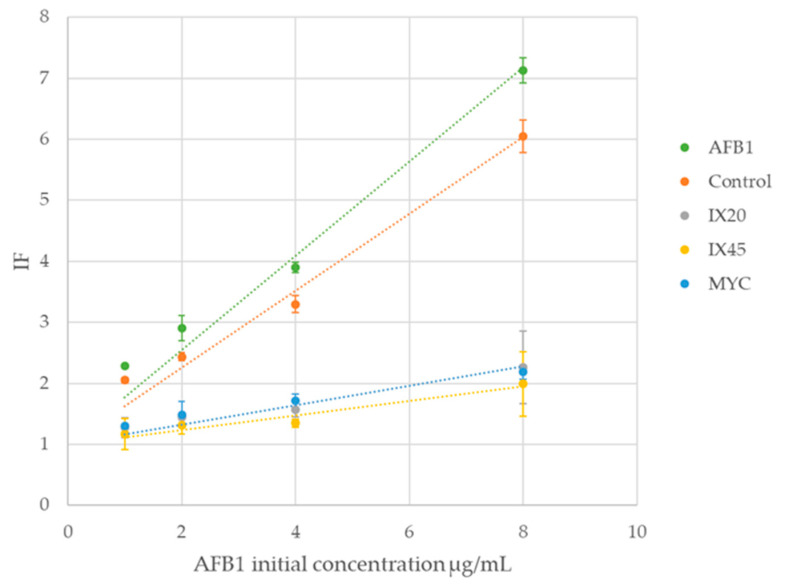
Plot of the SOS induction factors (IF) as a function of the initial concentration of AFB_1_ during degradation, and residual toxicity assays of *Streptomyces* isolates IX20 and IX45 and MYC strain after 12 days at 25 °C and 180 rpm. Control consisted of CYB medium without bacteria incubated under the same conditions. Results are compared to the plot of the IF values of the AFB_1_ standard at the initial concentrations.

The IF values for an AFB_1_ standard, the control and the samples were plotted as a function of the AFB_1_ initial concentration (Figure 7). The intrinsic degradation of the control during the incubation period, compared to the AFB_1_ curve, is observed, as well as the stronger decreases in genotoxicity caused by the *Streptomyces* isolates and MYC, compared to the control and to the reference AFB_1_ concentration. 

## 3. Discussion

In this study, fifty-nine *Streptomyces* isolates and the *Streptomyces griseoviridis* strain from the commercial product Mycostop^®^ were confronted *in vitro* with the mycotoxinogenic fungus *Aspergillus flavus*. The effect of *Streptomyces* isolates and their CFEs on fungal growth and aflatoxin-specific production was evaluated, in addition to their ability to degrade aflatoxin B_1_. A study on the effect of the same bacterial collection against the storage fungus *Penicillium verrucosum* and its ochratoxin A was previously conducted [29]. As the preceding work brought out isolates with promising features, the collection was evaluated against *A. flavus*, another post-harvest wheat pathogen [11]. A comparison between the effects observed on *A. flavus* and those previously published on *P. verrucosum* is presented in the Supplementary Materials in the form of three heatmaps, one for growth (S3), one for mycotoxin-specific production (S4) and the last one for mycotoxin degradation (S5). 

**Figure 8:**
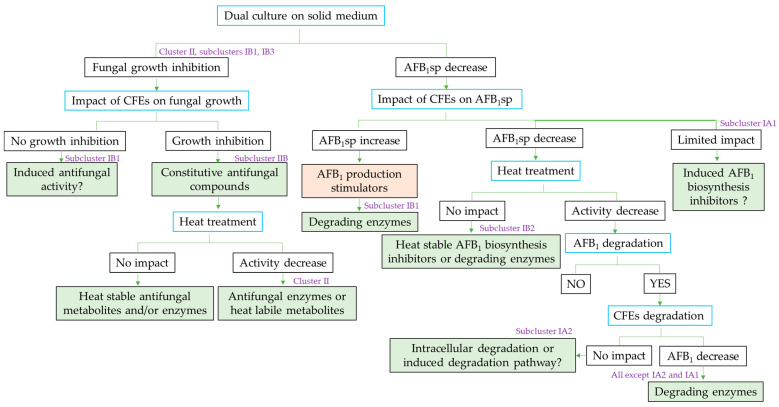
Workflow of the presented study for the preliminary identification of hypothetical antagonistic mechanisms of *Streptomyces* isolates against *Aspergillus flavus* development and AFB_1_ accumulation. Blue-colored rectangles indicate the different experimental stages, and filled rectangles (green, orange) indicate putative mechanisms. Examples of subclusters exhibiting the described mechanism are indicated in purple.

The research approach presented in this study is illustrated by the diagram in Figure 8, which constitutes a workflow towards the identification of the potential mechanisms involved in the interactions observed between the *Streptomyces* isolates and their metabolites with the fungus and its toxins. Clustering and correlation methods allowed highlighting isolates with promising detoxification capacities, as well as contrasting their mode of action against two saprophytic pathogens. This visualization, together with the generated workflow, constitutes a guideline for the selection of *Streptomyces* isolates according to the observed effects and the desired application. Furthermore, subsequent experiments, allowing the comprehension of interaction mechanisms between actinobacteria and fungi, will be based on the present general screening, in order to target the particular bioactive isolates identified during this work.

The first observation concerns the inhibition of fungal growth. *A. flavus* development was reduced by most *Streptomyces* isolates from cluster II during dual culture. The most promising isolates in cluster II for fungal inhibition include IX50 and IX23. Furthermore, CFEs were significantly less efficient at the tested concentration. However, the results obtained from both procedures are not quantitatively comparable, as only 10% of CFEs were tested in this assay, and the concentration in active metabolites and enzymes could differ from the dual culture experiment. Nevertheless, various hypotheses can be drawn from the literature. Firstly, the culture environment could be involved, as dual cultures were performed on solid CYA, while CFE production was conducted in liquid CYB. Indeed, it has been reported that aerial growth in solid media might impact *Streptomyces* metabolism [30]. The attenuated effect of CFEs in comparison to dual culture assays also suggests that bacterial antifungal metabolites and/or enzymes production could be positively induced by the presence of the fungus, as demonstrated by Wakefield et al. during co-culture of *Aspergillus fumigatus* and *Streptomyces leeuwenhoekii* [31]. Interestingly, bacterial isolates able to decrease *A. flavus* growth in dual culture were also able to reduce *P. verrucosum* development (Figure S3) [29], which implies that growth reduction could also partly originate from a general inhibition mechanism such as hydrolytic enzymes, pH variation or competition. For instance, the production of chitinase is frequently described in the literature for bacteria from the genus *Streptomyces* [12,32,33]. That being said, the heated CFEs’ efficacy was much lower than their non-heated counterparts at inhibiting fungal growth, which comforts the assumption that antifungal enzymes might be implied, produced mainly by isolates from cluster II. Furthermore, the remaining antifungal activity of the heated CFEs could be caused by the presence of thermally stable metabolites, as found by several research teams [34,35,36]. The pH increase due to *Streptomyces* metabolism, which, in our case, usually reaches 8.5 (data not shown), could also be at play in the growth inhibition observed. However, it cannot explain it fully as *A. flavus* growth in the 7–9 pH range is not strongly affected [37]. Finally, direct competition could also influence *A. flavus* growth [38,39], but the limited repartition and growth of *Streptomyces* isolates on the agar plate during our procedure are unlikely to lead to nutrient exhaustion and subsequent limitation of the fungal development. 

In a second step, the impact of *Streptomyces* isolates and their CFEs on mycotoxin production and degradation was evaluated. All isolates were able to decrease AFB_1_-specific production to at least 54% of the control during the dual culture assay, particularly IX07 and IX31, which diminished it almost entirely (0.71% and 0.83% of the control, respectively). Moreover, no aflatoxin overproduction was observed when the *Streptomyces* isolates were confronted in dual culture, which contrasts the previous results obtained with *P. verrucosum* (Figure S4), where strong fungal inhibitions were accompanied by a boost in OTA production [29]. The analysis of CFEs’ effect on AFB_1_sp, and the degradation abilities of the isolates and their CFEs, can help in highlighting the various mechanisms potentially involved in the observed reduction in AFB_1_ during the dual culture assays. Three main hypotheses can be mentioned, which may occur individually or concomitantly: the change in the culture environment (nutrient availability, pH), the presence of inhibitors of the AFB_1_ pathway and the degradation of the toxins by actinobacteria through intra- or extracellular enzymes. These three hypotheses will be discussed further regarding the results of this work and the available literature.

Some research indicates that sugar (as the main carbon source) and yeast extract utilization by aflatoxigenic fungi is linked to their aflatoxin biosynthesis [6,40]. Thus, if *Streptomyces* isolates limit the nutrients in the medium, making them less available for the fungi, this might provoke the general reduction in aflatoxin production during dual cultures. However, the fact that some CFEs strongly reduced AFB_1_sp (e.g., IX34, IX06) allows suggesting other mechanisms. For instance, *Streptomyces* are well known for their production of active molecules such as aflastatin A [20], blasticidin A [41] and dioctatin A [42], which were found to repress aflatoxin regulation genes, as proposed by Verheecke et al. [18]. Another study by Caceres et al. elucidated that the whole AFB_1_ gene cluster of *A. flavus* was downregulated, when exploring the impact of its dual culture with *S. roseolus* [22]. In cluster IA1, where the degradation abilities were weak as compared to the decrease in AFB_1_sp in the dual culture assay, a repression mechanism seems likely to have occurred. Furthermore, as mentioned before, most actinobacteria strains raise the pH in the culture medium during growth, which could also inhibit aflatoxin biosynthesis [43], as demonstrated by Keller et al., who found that 10-fold decreases in sterigmatocystin and aflatoxin production by *Aspergillus* spp. were caused at pH 8 [44]. Some CFEs greatly enhanced AFB_1_sp in cluster IB1 (IX54, IX05 and MYC), showing that some metabolites produced by *Streptomyces* isolates can stimulate the AFB_1_ pathway, possibly by triggering a defense mechanism from *A. flavus* [45,46,47]. This implicates that the metabolites produced by these isolates must not be applied for biocontrol purposes, as they may increase mycotoxin accumulation. However, they may be interesting for the study of mycotoxin elicitation [48,49]. A stimulation of aflatoxin biosynthesis by *Aspergillus flavus* due to bacterial biocontrol agents has been previously reported [50]. 

The majority of the studied *Streptomyces* isolates were able to degrade AFB_1_, either in solid and liquid media or by their CFEs. This is in agreement with the findings of Eshelli et al., whose degradation assays with three actinobacteria strains proved that all of them were able to significantly degrade AFB_1_ [14]. In general, aflatoxin degradation by actinobacteria has been demonstrated by several authors [26,51,52]. The observed reduction in aflatoxins by bacterial cells is unlikely to be due to cell wall binding, as the extraction protocol includes methanol and sonication, which are used for pellet washing and mycotoxin detachment [53]. In addition, previous research has shown that aflatoxin cell wall binding by *Streptomyces* is not significant in most cases [51]. Solid medium seems to slightly favor degradation, with an average reduction down to 33% of the control (43% for liquid medium). However, the degradation ability of bacteria cultivated in liquid and solid media remains strongly correlated (*r* = 0.8, *p* < 0.001). This suggests that the differentiated metabolism followed by *Streptomyces* isolates could be influenced by the culture environment [30,54] but is still rather similar in both conditions regarding AFB_1_ degradation. In contrast, degradation of OTA by the same *Streptomyces* isolates was scarce on solid medium, whereas it was largely spread in liquid medium (Figure S5) [29], which highlights the differences in the degradation mechanisms involved for both mycotoxins. Of note, *Streptomyces* isolates that were efficient at degrading OTA in liquid medium were also capable of degrading AFB_1_ in both liquid and solid environments (Figure S5). The strong AFB_1_ degradation capacity of numerous *Streptomyces* isolates such as MYC, IX28, IX20 and IX03 on solid media is probably one of the main mechanisms regarding the reduction in mycotoxin-specific production during dual cultures. The counterintuitive observation in cluster IB1 that CFEs could enhance AFB1sp accumulation, while the dual culture assay revealed a decrease in AFB1sp, can probably find its explanation in the strong degradation abilities found in this cluster.

A remarkable mechanism was involved in subcluster IA2, where the *Streptomyces* isolates and their CFEs were capable of decreasing the AFB_1_ amount without affecting *A. flavus* growth, a particularity that can be interesting for ecological niche preservation purposes. Yet, inhibition of fungal development can be desired during storage, as grain quality is strongly affected [55].

The fact that AFB_1_ was also degraded in CFEs points towards the presence of constitutive extracellular degrading enzymes, produced during submerged culture, rather than a potential binding to cells. This is comforted by the positive correlation between AFB_1_ degradation by *Streptomyces* isolates in liquid CYB and that by their CFEs, produced in CYB as well (*r* = 0.5, *p* = 0.002). AFB_1_ degradation by extracellular extracts of actinobacteria was already described by Alberts et al. when studying the degradation capacity of *Rhodococcus erythropolis* [56]. As elucidated by Taylor et al., aflatoxin-degrading actinobacteria are able to catalyze the reduction of the double bond of the α,β-unsaturated ester moiety of aflatoxins thanks to enzymes using the F_420_H_2_ deazaflavin cofactor, leading to spontaneous hydrolysis and detoxification [57]. This mechanism could be widespread among actinobacteria, as proposed by Lapalikar et al. [58], and it might apply to the isolates in this study. The capacity of nearly all of the tested CFEs to degrade AFB_1_ opens new perspectives for further research to isolate and identify the degrading enzymes involved, and to determine the mechanisms at play. Isolates IX09, MYC and IX45 seem particularly interesting for this purpose. 

As already observed for antifungal activity, thermal treatment of CFEs resulted in weakened detoxification capacities, suggesting the presence of labile mycotoxin-degrading enzymes or metabolites. However, the activity of heated CFEs was not completely eliminated, which may indicate the presence of heat-resistant compounds and/or enzymes. A complementary method for the confirmation of enzymatic activities in CFEs could be the use of proteinase K, as described by Guan et al. when evaluating the cell-free extract of *Stenotrophomonas maltophilia* [59]. This will help in confirming the results obtained with CFE heat inactivation, as some AFB_1_-degrading enzymes have been found to be heat-stable [36,60]. Finally, a comparison between the activities of intra- and extracellular extracts could help to identify the location of the mycotoxin-degrading compounds [61]. These two approaches could be applied to a selection of isolates for an in-depth investigation of the mechanism of degradation and for the subsequent isolation of active compounds, as demonstrated by several research groups [57,59,62,63]. 

A common bacterial degradation mechanism of AFB_1_ consists in the cleavage of the lactone ring, which may lead to the formation of aflatoxin D_1_ and D_2_ [14,64]. This cleavage is accompanied by a loss of fluorescence, meaning that these compounds cannot be detected by fluorescence HPLC [65]. As no fluorescent breakdown products were observed in our assay, a further search for degradation by-products was conducted by HPLC-MS with two major degrading isolates (IX20, IX45) and MYC. During the search for AFB_1_ degradation by-products, the peaks of two unknown compounds were observed only for the MYC MS spectra, corresponding to a first compound of formula C_10_H_10_NO (*m/z* 160.0770) and to a second of formula C_10_H_10_NO_2_ (*m/z* 176.0720). These molecules are neither present in the mass spectrum of the control nor in the samples grown without AFB_1_ addition, which suggests that they were produced during AFB_1_ degradation. Additionally, they do not correspond to an already described degradation by-product, and the mechanism of their production was not elucidated. Several unknown breakdown products were identified by Iram et al. when studying the degradation of AFB_1_ and AFB_2_ by *Corymbia citriodora* [66], by *Ocimum basilicum* and *Cassia fistula* [67] and by *Trachyspermum ammi* [68]. Similarly, some residual products of AFB_1_ degradation were described by Eshelli et al., namely, C_17_H_15_O_7_ (*m/z* 331.2845), C_16_H_15_O_5_ (*m/z* 287.2220) and C_13_H_17_O_4_ (*m/z* 237.1123), found after 72 h of incubation with actinobacteria [14]. The by-products discovered by Iram et al. and Eshelli et al. have a higher molecular weight than the compounds found in this study; this may suggest that after 12 days of culture, known degradation by-products could further be degraded into smaller unknown molecules, or it may suggest the occurrence of a different degradation mechanism. For IX20 and IX45, no degradation by-products were detected in both extraction solvents. This finding is similar to that of Alberts et al., who were unable to detect degradation by-products by HPLC-MS when studying aflatoxin degradation by *Rhodococcus erythropolis* [56]. Regarding the presence of an AFB_1_ isomer—also observed for the control—no clear explanation was found, other than the theory of a transitional conformation variation during the incubation.

Residual toxicity evaluation by the SOS chromotest showed that the three isolates tested were able to decrease the genotoxicity of the sample down to about 30% compared to the control. Harkai et al. determined that *Streptomyces cacaoi subsp. asoensis* was able to reduce the genotoxicity of AFB_1_, at an initial concentration of 1 µg/mL, as it decreased the SOS induction factor from 2.25 to 1.37 [51]. In the present study, an initial concentration of 4 µg/mL of AFB_1_ could be detoxified from an initial IF of 3.9 to a non-genotoxic level (IF < 1.5) by the *Streptomyces* isolate IX45, while isolates IX20 and MYC could eliminate the genotoxicity of an initial AFB_1_ concentration of ~3 µg/mL (IF = 3.32). However, the percentage of decrease in genotoxicity was slightly lower than the percentage of reduction in AFB_1_, which was 87% for IX20, 96% for IX45 and 75% for MYC. This could indicate that even if the aflatoxin is degraded, some resulting by-products might remain genotoxic to a certain degree, which emphasizes the importance of the verification of the residual toxicity.

## 4. Conclusions

In conclusion, several *Streptomyces* isolates were able to inhibit *Aspergillus flavus* growth, and most importantly, the entire collection was able to decrease the amount of aflatoxin B_1_ accumulated during fungal growth, and also to degrade the mycotoxin, at different rates. Most CFEs also decreased the aflatoxin amount, either during fungal growth or by posterior degradation, which is promising for the research and isolation of active enzymes and metabolites. As *Streptomyces* strains are not classified as GRAS (Generally Recognized as Safe) microorganisms, their direct application is not authorized. Nevertheless, they constitute a promising source of mycotoxin-degrading enzymes or mycotoxin metabolic pathway inhibitors for the detoxification of food matrices. A verification of residual toxicity during detoxification assays is imperative to guarantee that the resulting by-products do not represent any risk. The approach presented in this study allows the evaluation of potential biocontrol agents by their antifungal and detoxifying capacities, as well as their clustering and classification according to common features and the comparison to their effect on different parameters. More importantly, this procedure leads to the identification and selection of promising candidates for subsequent research. The dissimilarities in the effect of *Streptomyces* isolates on AFB_1_ and OTA could be related to several factors such as the different degradation mechanisms involved, distinct enzymes with different optimal conditions and the particular functions of each mycotoxin for the fungus. Further studies on the bioactive isolates identified during this work will allow a better understanding of these mechanisms. 

## 5. Materials and Methods

### 5.1. Streptomyces Isolates

Fifty-nine *Streptomyces* isolates were retrieved from organic amendments and soil samples collected at the Hérault department in the South of France. A 16S preliminary identification demonstrated that all the isolates in the collection belonged, at 97%, to the genus *Streptomyces* (data not shown). The isolates constitute a collection maintained in the laboratory of the UMR QualiSud at the University of Montpellier. Mycostop^®^ (MYC) strain *Steptomyces griseoviridis* K61 was included in the tests as a commercialized biocontrol agent for comparison. The isolates were grown on ISP4 medium (10 g/L starch, 1 g/L K_2_HPO_4_, 1 g/L MgSO_4_, 2 g/L (NH_4_)_2_SO_4_, 1 g/L CaCO_3_, 1 mg/L FeSO_4_, 1 mg/L MgCl_2_, 1 mg/L ZnSO_4_, 18 g/L bacteriological agar) for 11 days at 28°C. Spores were then collected by scraping the surface of the Petri dish with 5 mL of distilled water +0.01% Tween 20 and filtered through sterile cotton. The spore suspensions were aliquoted and then stored at −80 °C. 

### 5.2. Streptomyces Isolates Spore Numeration by Flux Cytometer

For *Streptomyces* isolates spore count, a Novocyte ACEA flux cytometer was employed. After counting, the results were validated by colony-forming units on CYA medium after 11 days of culture. Before each test, bacterial spore suspensions were unfrozen and diluted to a concentration of 10^7^ spores/mL.

### 5.3. Pathogen Strains

A mycotoxinogenic strain of *Aspergillus flavus*, E73/NRRL62477, kindly provided by Dr. Olivier Puel from INRAE’s UMR Toxalim, was employed to carry out this work. The fungus was maintained on PDA (Biokar BK095HA) inclined tubes covered in paraffin oil prior to use. For fungal spore harvesting, the pathogen was grown on PDA plates for 7 days at 25 °C, and then spores were scraped from the surface by adding 10 mL of distilled sterile water and filtered through sterile cotton. Pathogen spores were harvested and enumerated before each test.

### 5.4. Antagonistic Evaluation In Vitro

#### 5.4.1. Streptomyces Isolate Cells in Dual Cultures on Solid Medium

Antagonistic assays were performed on Czapek Yeast Agar medium (CYA: 30 g/L sucrose, 5 g/L yeast extract, 1 g/L K_2_HPO_4_, 0.3 g/L NaNO_3_, 0.05 g/L KCl, 0.05 g/L MgSO_4_, 1 mg/L FeSO_4_, 1 mg/L ZnSO_4_, 0.5 mg/L CuSO_4_, 15 g/L agar, pH ~7.4) which allowed both proper pathogen and bacteria growth and sporulation. 

Dual culture assays were implemented on Petri dishes by inoculating 10 µL of the bacterial isolate spore suspension at 10^7^ CFU/mL on each side of the plate. After three days at 25 °C, 10 µL of the pathogen spore suspension at 10^6^ CFU/mL was inoculated at the center of the plate and left for 5 more days at 25 °C before image analysis and mycotoxin extraction. As a control, the pathogen was inoculated on a CYA plate without bacteria. All tests were conducted in triplicate. 

#### 5.4.2. Cell-Free Extracts of Liquid Cultures

*Streptomyces* isolates were cultured on Czapek Yeast Extract broth (CYB: 30 g/L sucrose, 5 g/L yeast extract, 1 g/L K_2_HPO_4_, 0.3 g/L NaNO_3_, 0.05 g/L KCl, 0.05 g/L MgSO_4_, 1 mg/L FeSO_4_, 1 mg/L ZnSO_4_, 0.5 mg/L CuSO_4_, pH ~7.4) medium for 5 days at 25 °C and 180 rpm. Then, liquid cultures were centrifuged at 10,000× *g* for 5 min, and the supernatant was filtered through a PES filter at 0.22 µm to eliminate bacterial cells. The resulting cell-free extracts (CFE) were used to evaluate the effect of bacterial metabolites on the fungal pathogen’s growth and mycotoxin production on CYA for 8 days at 25 °C. In order to identify enzymatic activities, CFEs were also tested after a heat treatment at 100 °C for 10 min.

#### 5.4.3. Pathogen Surface Growth Measurement and AFB_1_-Specific Production Calculation

Surface growth of *A. flavus* colonies was measured in ImageJ software (1.52a, Wayne Rasband National Institute of Health, Bethesda, MD, USA, 2018). Growth inhibition was established in comparison with the control without bacteria which represented 100% of growth. Results are given in % of control.

(1) $$Growth\;ratio\left(\%\;of\;control\right)=\frac{Assay\;area\;\left(cm^{2}\right)}{Control\;area\;\left(cm^{2}\right)\;}\times 100$$

*AFB*_1_-specific production (*AFB*_1_*sp*) was calculated by dividing the mycotoxin amount in the whole sample by the area of the fungal colony. This calculation allows comparing the mycotoxin accumulation in the medium normalized to the fungal surface.

(2) $$AFB_{1}\;specific\;production\;\left(AFB_{1}sp\right)\;\;\;\;\;\;\;\;\;\;\;\;\;\;\;\;=\frac{Amount\;of\;mycotoxin\;produced\;\left(ng\right)}{Colony\;area\;\left(cm^{2}\right)}$$

The percentage of reduction for *AFB*_1_-specific production was calculated in comparison to the control without bacteria.

(3) $$AFB_{1}{}_{sp}\;ratio\;\left(\%\;of\;control\right)=\frac{AFB_{1}{}_{sp}\;assay}{AFB_{1}{}_{sp}\;control}\times 100$$

### 5.5. Mycotoxin Degradation Assay

#### 5.5.1. Screening of AFB_1_ Degradation by *Streptomyces* Isolates and Their CFEs

Degradation assays were performed using Aflatoxin B_1_ (Sigma Aldrich, St. Quentin Fallavier, France and Libios, Vindry sur Turdine, France) suspended in acetonitrile in order to prepare a stock solution of 1 mg/mL. Control concentration was 2 µg/mL. Mycotoxin was added to solid CYA and liquid CYB media, followed by the addition of 20 µL of bacterial isolate spore suspension at 10^7^ spores/mL, and incubated at 25 °C with an agitation at 180 rpm for the liquid cultures. After 10 days on solid medium and 5 days in liquid medium, mycotoxin was extracted and analyzed as further described. CFEs, produced as described before, were also tested for their ability to degrade AFB_1_ after being in contact with the mycotoxin for 48 h at 25 °C and 180 rpm. As control, mycotoxin was spiked in solid CYA or liquid CYB medium and incubated following the same incubation protocols as the samples.

(4) $$AFB_{1}\;degradation\;\left(\%\;of\;control\right)=\frac{AFB_{1}{}_{ng/ml}\;assay}{AFB_{1}{}_{ng/ml}\;control}\times 100$$

#### 5.5.2. Degradation Assays for the Search of Breakdown Products

Three isolates with strong degrading abilities in liquid medium (IX20, IX45 and MYC) were selected to perform additional degradation assays. For this, they were cultured for 12 days in 20 mL of CYB medium containing 8 µg/mL of AFB_1_ inoculated with 100 µL of a bacterial spore suspension at 1 × 10^7^ spores/mL and incubated for 12 days at 25 °C and 180 rpm. The controls consisted of CYB medium with the same concentration of AFB_1_ and without the bacteria inoculum, and bacteria cultured in CYB without AFB_1_, both incubated under the same conditions as the main test. These controls allowed subtracting the spectra belonging to bacterial metabolites and culture medium from the spectra obtained during degradation, in order to identify the remaining peaks. All tests were conducted in triplicate. Several known breakup products of AFB_1_ degradation such as aflatoxicol, aflatoxin B_2_a and D aflatoxins were searched on HPLC-MS.

### 5.6. Mycotoxin and Degradation By-Products Analysis

#### 5.6.1. Sample Extraction 

For the mycotoxin extraction of assays with antagonistic isolates and CFEs, the whole content of the Petri dish was transferred into a plastic container and then cut into small pieces with a scalpel. After weighing the agar, 100 mL of acidified methanol (3.85% formic acid) was added to the sample, followed by 20 min of agitation at 250 rpm. An amount of 500 µL of the mixture was evaporated in a Speed Vac (Eppendorf^®^ AG, Hamburg, Germany) at 60 °C until dryness, and then 2 mL of the mobile phase (water/methanol 55:45 (*v*/*v*) with 119 mg Kbr (0.001 M Kbr) and 350 µL of 4M nitric acid) was added. To ensure that mycotoxin was not bound by the cells, samples underwent 20 min of sonication, followed by 10 min in a cell disruptor, and, finally, were thoroughly mixed in a vortex before filtering through a 0.45 µM PTFE filter into a glass vial for HPLC analysis.

For the search of degradation by-products, the resulting supernatants were mixed with equivalent volumes of chloroform or ethyl acetate by agitating vigorously for 20 min. Then, the organic phase was retrieved and filtered through a 0.45 µM PTFE filter into a glass vial for HPLC-MS analysis.

#### 5.6.2. AFB_1_ Quantification by Fluorescence HPLC

AFB_1_ was quantified by HPLC using a fluorescence detector (Shimadzu RF 20A, Japan) after post-column electrochemical derivatization (Kobra Cell™ R. Biopharm Rône Ltd., Glasgow, UK). The operating conditions were as follows: injection volume of 100 µL; C18 reverse-phase HPLC column, Uptisphere type 5 ODB, ODS, 5 µm particle size, 5 ODB, 250 × 4.6 mm, with identical pre-column, thermostatically controlled at 40 °C; isocratic flow rate of 0.8 mL/min (water/methanol 55:45 (*v*/*v*) with 119 mg Kbr (0.001 M Kbr) and 350 µL of 4M nitric acid). Excitation wavelength was 360 nm, and emission wavelength was 450 nm. The concentrations were calculated from a calibration curve established from an AFB_1_ standard (25 µg/mL; Biopharm Rhône Ltd., Glasgow, UK) [69]. Detection and quantification limits were established at 0.05 and 0.2 ng/mL, respectively. 

#### 5.6.3. Search for Degradation By-Products by HPLC-MS

For the search of degradation by-products, the chromatographic equipment consisted of an Acquity H-Class UPLC (Waters, Milford) equipped with a quaternary pump, solvent degasser and thermostatted column compartment. A reversed-phase column was used for separation: Kinetex C18 (2.1 × 100 mm 1.7 μm from Phenomenex). Mobile phases A and B consisted of water (0.1% formic acid) and acetonitrile (0.1% formic acid), respectively. The 15 min linear gradient program used was 5–50% B for 8 min, then 50–100% B for 3 min and decreasing from 100 to 10% B for 0.1 min, followed by a 3.9-min post-run isocratic step at 5% B to re-equilibrate the column. The flow rate was constant at 0.5 mL/min at 25 °C.

Mass spectrometry detections were carried out using the Waters UPLC system described above coupled to a Synapt G2-S mass spectrometer (Waters, Milford) operating in positive ion mode. Mass spectra were recorded between 50 and 2000 Da. The following parameters were used for all experiments: capillary voltage 3 kV; sampling cone 20 V; desolvation temperature 450 °C; source temperature 140 °C.

### 5.7. Evaluation of Residual Toxicity by SOS Chromotest 

The remaining liquid cultures of the two isolates (IX20, IX45) and MYC, selected for the research of degradation by-products, were analyzed to determine if the degradation leads to an effective detoxification of the mycotoxin. At the end of the incubation period, a fraction of the samples was extracted as described previously and analyzed by fluorescence HPLC to determine the decrease in AFB_1_ and by HPLC-MS for the search for degradation by-products. Then, the remaining fraction of the liquid culture was filtered through a hydrolyzed PTFE filter to obtain cell-free extracts (CFEs), which were evaluated for residual toxicity using the SOS chromotest.

The SOS chromotest is a colorimetric assay based on the response of a mutant strain of *Escherichia coli* PQ37 to the DNA damage caused by genotoxic compounds. In order to differentiate direct and indirect genotoxic activities, the test uses S9 rat liver homogenate to identify compounds that require metabolic bioactivation to exhibit a genotoxic reaction. In addition to the measure of β-galactosidase induction, the SOS chromotest allows measuring cell survival by means of alkaline phosphatase as a toxicity assay [26,27,28]. The SOS-Chromotest Kit was purchased from Environmental Bio-Detection Products Inc., Canada. 

Preliminary tests (not shown) allowed us to determine that the minimal concentration of AFB_1_ needed to trigger a genotoxic effect on the *E. coli* test strain was 2 µg/mL. Thus, AFB_1_ concentration for the search for degradation by-products and the posterior evaluation of residual toxicity was increased to 8 µg/mL.

The assay was performed on 96-well flat-bottom plates, using an Enspire Multimode plate reader (Perkin Elmer, Waltham, MA, USA). Positive controls for direct and indirect genotoxicities were, respectively, 4-nitroquinoline 1-oxide (4-NQO) and 2-amino-anthracene (2-AA), in two-fold dilutions. The background and negative control contained 10% DMSO (dimethyl sulfoxide) in sterile 0.85% saline. Ten microliters of the CFEs obtained as described before was transferred to the corresponding wells to obtain a series of two-fold dilutions. Finally, 100 µL of an overnight culture of the test *E. coli* strain (final OD_600_ = 0.5) was added to the control and degradation assay samples and incubated for 2 h until color development. After the incubation period, absorbances were read at 600 nm to determine β-galactosidase production and at 420 nm to determine viability of the bacteria.

For the analysis of the results, the induction factor (IF) was calculated as follows:

(5) $$IF=\frac{\left(OD_{600,\;i}-OD_{600,\;B}\right)/\left(OD_{600,\;N}-OD_{600,\;B}\right)}{\left(OD_{420,i}-OD_{420,B}\right)/\left(OD_{420,\;N}-OD_{420,B}\right)}$$

where:

$OD_{600,\;i}=$ absorbance at 600 nm for sample wells;
$OD_{600,\;B}=$ average absorbance at 600 nm for reagent blank wells;
$OD_{600,\;N}=$ absorbance at 600 nm for negative control wells;
$OD_{420,\;i}=$ absorbance at 420 nm for sample wells;
$OD_{420,\;B}=$ average absorbance at 420 nm for reagent blank wells;
$OD_{420,\;N}=$ absorbance at 420 nm for negative control wells.

As a general classification, an SOSIF lower than 1.5 is not genotoxic, between 1.5 and 2 is inconclusive and higher than 2 is genotoxic. 

### 5.8. Data Analysis and Data Visualization

Preprocessing of the data for the heatmap consisted of a conversion of percentages to Z-scores calculated as follows: Z−score=(X−μ)/σ, where X is the measurement, μ is the mean value of the effect of all isolates in a category (growth, mycotoxin-specific production, mycotoxin degradation) and σ is the standard deviation of the value of the effect of all isolates in a category. Boxplots and Pearson correlation graphics were developed in Rstudio.

## Figures and Tables

**Table 1:** Summary of specific features observed during the clustering analysis of the impact of the screened *Streptomyces* isolates and their cell-free extracts (CFEs) on *Aspergillus flavus* growth and aflatoxin B_1_-specific production (AFB_1_sp), together with their ability to degrade AFB_1_.

Cluster	Specific Features	Subcluster		Specific Features	Isolate/Strain
		**A**	**1**	Limited inhibition of *A. flavus* growth. Moderate decrease in AFB_1_sp in dual culture. Limited degradation capacity.	IX16, IX37, IX38, IX47, IX53, IX57
**I**	Moderate effect on *A. flavus* growth. Contrasted effect on AFB_1_-specific production and its degradation.		**2**	Lack of inhibition of *A. flavus* growth. Strong decrease in AFB_1_sp in dual culture. Moderate capacity of CFEs to decrease AFB_1_sp. Moderate degradation capacity of bacterial cells.	IX30, IX40, IX41, IX43, IX44
		**B**	**1**	Moderate decrease in AFB_1_sp in dual culture. Strong increase in AFB_1_sp provoked by CFEs. Strong degradation capacity of bacterial cells.	IX05, IX54, MYC
			**2**	Moderate decrease in AFB_1_sp. Strong degradation capacity of bacterial cells.	IX01, IX03, IX04, IX12, IX20, IX25, IX28, IX35, IX36, IX39, IX45, IX58
			**3**	Strong decrease in AFB_1_sp in dual culture and by unheated CFEs. Moderate degradation capacities.	IX02, IX06, IX08, IX29, IX33, IX34, IX42, IX46
**II**	Strong inhibition of *A. flavus* growth (dual culture). Strong effect on AFB_1_sp. Moderate degradation by bacterial cells and CFEs.	**A**		Strong inhibition of *A. flavus* growth in dual culture. Strong decrease in AFB_1_sp in dual culture. Moderate degradation by bacterial cells and by CFEs.	IX14, IX22, IX23, IX48, IX50, IX55, IX56, IX59
		**B**		Strong inhibition of *A. flavus* growth in dual culture. Strong decrease in AFB_1_sp in dual culture. Moderate decrease in AFB_1_sp by CFEs. Moderate degradation by bacterial cells and by CFEs.	IX07, IX09, IX10, IX11, IX13, IX15, IX17, IX18, IX19, IX21, IX24, IX26, IX27, IX31, IX32, IX49, IX51, IX52

**Table 2:** Evaluation of the residual toxicity of CYB supernatants issued from the degradation of 8 µg/mL of AFB_1_ by *Streptomyces* isolates IX20 and IX45 and MYC strain after 12 days at 25 °C and 180 rpm.

			*Streptomyces* Isolate/Strain Reduction in Genotoxicity		
		
	Standard AFB_1_	Control	IX20	IX45	MYC
Residual AFB_1_ (µg/mL)	8	7.71 ± 0.22	0.98 ± 0.09	0.32 ± 0.04	1.95 ± 1.65
IF	7.13	6.05 ± 0.27	2.26 ± 0.60	1.99 ± 0.53	2.18 ± 0.11
% IF	100	85 ± 4	32 ± 8	28 ± 7	31 ± 2

## Supplementary Materials

The following are available online at https://www.mdpi.com/article/10.3390/toxins13050340/s1; Figure S1: Heatmap of the effect of *Streptomyces* isolates and their CFEs on *Aspergillus flavus* growth (Category 1) and AFB1 accumulation (Category 2), along with the bacteria’s ability to degrade AFB1 in solid and liquid medium and by their CFEs (Category 3). Results are given in a range of colours according to their Z-score, where purple to black represents a lack of activity or an in-crease compared to the control, whereas yellow represents the strongest activity, Figure S2: Pear-son’s correlation of the effects of *Streptomyces* isolates and their CFEs on *Aspergillus flavus* growth and AFB1 accumulation, along with the degradation by bacteria in solid and liquid medium, and the degradation by their CFEs, Table S1: Values of the Pearson correlation (r) between the meas-ured parameters during the screening of the effect of *Streptomyces* isolates and Mycostop® and their CFEs on *A. flavus* growth and AFB1 accumulation, Figure S3: Heatmap of the effect of *Streptomyces* isolates and their CFEs on *Aspergillus flavus* (A) and *Penicillium verrucosum* (P) growth, Figure S4: Heatmap of the effect of *Streptomyces* isolates and their CFEs on AFB1 accumulation by *Aspergillus flavus* and on OTA accumulation by *Penicillium verrucosum*, Figure S5: Heatmap of *Streptomyces* isolates and their CFEs ability to degrade AFB1 and OTA.

## References

[1] Oliveira C.A.F., Bovo F., Humberto C., Vincenzi A., Ravindranadha K. (2013). Recent Trends in Microbiological Decontamination of Aflatoxins in Foodstuffs. Aflatoxins—Recent Advances and Future Prospects.

[2] McCormick S.P. (2013). Microbial Detoxification of Mycotoxins. J. Chem. Ecol..

[3] Riba A., Bouras N., Mokrane S., Mathieu F., Lebrihi A., Sabaou N. (2010). *Aspergillus* section *Flavi* and aflatoxins in Algerian wheat and derived products. Food Chem. Toxicol..

[4] Masood M., Iqbal S.Z., Asi M.R., Malik N. (2015). Natural occurrence of aflatoxins in dry fruits and edible nuts. Food Control.

[5] Azzoune N., Mokrane S., Riba A., Bouras N., Verheecke C., Sabaou N., Mathieu F. (2016). Contamination of common spices by aflatoxigenic fungi and aflatoxin B1 in Algeria. Qual. Assur. Saf. Crop. Foods.

[6] Yu J. (2012). Current understanding on aflatoxin biosynthesis and future perspective in reducing aflatoxin contamination. Toxins.

[7] Agriopoulou S., Stamatelopoulou E., Varzakas T. (2020). Advances in occurrence, importance, and mycotoxin control strategies: Prevention and detoxification in foods. Foods.

[8] European Commission (2016). RASFF—Food and Feed Safety Alerts|Food Safety. https://ec.europa.eu/food/safety/rasff_en.

[9] Battilani P., Toscano P., Van Der Fels-Klerx H.J., Moretti A., Leggieri M.C., Brera C., Rortais A., Goumperis T., Robinson T. (2016). Aflatoxin B 1 contamination in maize in Europe increases due to climate change. Sci. Rep..

[10] European Commission Maximum Levels for Certain Contaminants in Foodsuffs. https://eur-lex.europa.eu/legal-content/EN/ALL/?uri=celex:32006R1881.

[11] Birck N.M.M., Lorini I., Scussel V.M. Fungus and mycotoxins in wheat grain at post harvest. Proceedings of the 9th International Working Conference on Stored Product Protection.

[12] Ren X., Zhang Q., Zhang W., Mao J., Li P. (2020). Control of aflatoxigenic molds by antagonistic microorganisms: Inhibitory behaviors, bioactive compounds, related mechanisms, and influencing factors. Toxins.

[13] Wu Q., Jezkova A., Yuan Z., Pavlikova L., Dohnal V., Kuca K. (2009). Biological degradation of aflatoxins. Drug Metab. Rev..

[14] Eshelli M., Harvey L., Edrada-Ebel R., McNeil B. (2015). Metabolomics of the bio-degradation process of aflatoxin B1 by actinomycetes at an initial pH of 6.0. Toxins.

[15] Verheecke C., Liboz T., Mathieu F. (2016). Microbial degradation of aflatoxin B1: Current status and future advances. Int. J. Food Microbiol..

[16] Barka E.A., Vatsa P., Sanchez L., Gaveau-Vaillant N., Jacquard C., Klenk H.-P., Clément C., Ouhdouch Y., Van Wezel G.P. (2016). Taxonomy, Physiology, and Natural Products of Actinobacteria. Microbiol. Mol. Biol. Rev..

[17] Verheecke C., Liboz T., Anson P., Zhu Y., Mathieu F. (2015). *Streptomyces–Aspergillus flavus* interactions: Impact on aflatoxin B accumulation. Food Addit. Contam. Part A.

[18] Verheecke C., Liboz T., Darriet M., Sabaou N., Mathieu F. (2014). In vitro interaction of actinomycetes isolates with *Aspergillus flavus*: Impact on aflatoxins B1 and B2 production. Lett. Appl. Microbiol..

[19] Wongsariya K., Thawai C. (2019). Antifungal Activity against the Growth of Aflatoxin Producing Fungi from Soil Actinobacteria. J. Adv. Agric. Technol..

[20] Sakuda S., Ono M., Furihata K., Nakayama J., Suzuki A., Isogai A. (1996). Aflastatin A, a novel inhibitor of aflatoxin production of *Aspergillus parasiticus*, from *Streptomyces*. J. Am. Chem. Soc..

[21] Sakuda S. (2010). Mycotoxin production inhbitors from natural products. Mycotoxins.

[22] Caceres I., Snini S.P., Puel O., Mathieu F. (2018). *Streptomyces roseolus*, A Promising Biocontrol Agent against *Aspergillus flavus*, the Main Aflatoxin B1 Producer. Toxins.

[23] Vanhoutte I., Audenaert K., De Gelder L. (2016). Biodegradation of mycotoxins: Tales from known and unexplored worlds. Front. Microbiol..

[24] Adebo O.A., Njobeh P.B., Gbashi S., Nwinyi O.C., Mavumengwana V. (2017). Review on microbial degradation of aflatoxins. Crit. Rev. Food Sci. Nutr..

[25] Vincenzi A., Silva F., Naira L., Oliveira C.A.F. (2011). Biomarkers of Aflatoxin Exposure and Its Relationship with the Hepatocellular Carcinoma. Aflatoxins—Biochemistry and Molecular Biology.

[26] Krifaton C., Kriszt B., Szoboszlay S., Cserháti M., Szűcs Á., Kukolya J. (2011). Analysis of aflatoxin-B1-degrading microbes by use of a combined toxicity-profiling method. Mutat. Res. Genet. Toxicol. Environ. Mutagen..

[27] Quillardet P., De Bellecombe C., Hofnung M. (1985). The SOS Chromotest, a colorimetric bacterial assay for genotoxins: Validation study with 83 compounds. Mutat. Res. Mutagen. Relat. Subj..

[28] Quillardet P., Hofnung M. (1993). The SOS chromotest: A review. Mutat. Res. Rev. Genet. Toxicol..

[29] Campos-Avelar I., De La Noue A.C., Durand N., Fay B., Martinez V., Fontana A., Strub C., Schorr-Galindo S. (2020). Minimizing ochratoxin a contamination through the use of actinobacteria and their active molecules. Toxins.

[30] Manteca A., Yague P. (2018). *Streptomyces* differentiation in liquid cultures as a trigger of secondary metabolism. Antibiotics.

[31] Wakefield J., Hassan H.M., Jaspars M., Ebel R., Rateb M.E. (2017). Dual induction of new microbial secondary metabolites by fungal bacterial co-cultivation. Front. Microbiol..

[32] Nazari B., Saito A., Kobayashi M., Miyashita K., Wang Y., Fujii T. (2011). High expression levels of chitinase genes in *Streptomyces* coelicolor A3(2) grown in soil. FEMS Microbiol. Ecol..

[33] Williamson N., Brian P., Wellington E. (2000). Molecular detection of bacterial and streptomycete chitinases in the environment. Antonie Van Leeuwenhoek.

[34] Prapagdee B., Kuekulvong C., Mongkolsuk S. (2008). Antifungal potential of extracellular metabolites produced by *Streptomyces hygroscopicus* against phytopathogenic fungi. Int. J. Biol. Sci..

[35] Boukaew S., Prasertsan P. (2014). Suppression of rice sheath blight disease using a heat stable culture filtrate from *Streptomyces philanthi* RM-1-138. Crop Prot..

[36] Sangare L., Zhao Y., Folly Y.M.E., Chang J., Li J., Selvaraj J.N., Xing F., Zhou L., Wang Y., Liu Y. (2014). Aflatoxin B₁ degradation by a *Pseudomonas* strain. Toxins.

[37] Wheeler K.A., Hurdman B.F., Pitt J. (1991). Influence of pH on the growth of some toxigenic species of *Aspergillus*, *Penicillium* and *Fusarium*. Int. J. Food Microbiol..

[38] Nguyen P.-A., Strub C., Fontana A., Schorr-Galindo S. (2017). Crop molds and mycotoxins: Alternative management using biocontrol. Biol. Control.

[39] Olivier P., Marzin D. (1987). Study of the genotoxic potential of 48 inorganic derivatives with the SOS chromotest. Mutat. Res. Toxicol..

[40] Davis N.D., Diener U.L., Agnihotri V.P. (1967). Production of aflatoxins B1 and G1 in chemically defined medium. Mycopathol. Mycol. Appl..

[41] Yoshinari T., Noda Y., Yoda K., Sezaki H., Nagasawa H., Sakuda S. (2010). Inhibitory activity of blasticidin A, a strong aflatoxin production inhibitor, on protein synthesis of yeast: Selective inhibition of aflatoxin production by protein synthesis inhibitors. J. Antibiot..

[42] Yoshinari T., Akiyama T., Nakamura K., Kondo T., Takahashi Y., Muraoka Y., Nonomura Y., Nagasawa H., Sakuda S. (2007). Dioctatin A is a strong inhibitor of aflatoxin production by *Aspergillus parasiticus*. Microbiology.

[43] Woloshuk C.P., Shim W.B. (2013). Aflatoxins, fumonisins, and trichothecenes: A convergence of knowledge. FEMS Microbiol. Rev..

[44] Keller N.P., Nesbitt C., Sarr B., Phillips T.D., Burow G.B. (1997). pH regulation of sterigmatocystin and aflatoxin biosynthesis in *Aspergillus* spp.. Phytopathology.

[45] Pfliegler W.P., Pócsi I., Győri Z., Pusztahelyi T. (2020). The *Aspergilli* and Their Mycotoxins: Metabolic Interactions With Plants and the Soil Biota. Front. Microbiol..

[46] Van Rij E.T., Girard G., Lugtenberg B.J.J., Bloemberg G.V. (2005). Influence of fusaric acid on phenazine-1-carboxamide synthesis and gene expression of *Pseudomonas chlororaphis* strain PCL1391. Microbiology.

[47] Rasmussen T.B., Skindersoe M.E., Bjarnsholt T., Phipps R.K., Christensen K.B., Jensen P.O., Andersen J.B., Koch B., Larsen T.O., Hentzer M. (2005). Identity and effects of quorum-sensing inhibitors produced by *Penicillium* species. Microbiology.

[48] Sweany R.R., Damann K.E. (2020). Influence of Neighboring Clonal-Colonies on Aflatoxin Production by *Aspergillus flavus*. Front. Microbiol..

[49] Medina A., Mohale S., Samsudin N.I.P., Rodriguez-Sixtos A., Rodriguez A., Magan N. (2017). Biocontrol of mycotoxins: Dynamics and mechanisms of action. Curr. Opin. Food Sci..

[50] Al-Saad L.A., Al-Badran A.I., Al-Jumayli S.A., Magan N., Rodríguez A. (2016). Impact of bacterial biocontrol agents on aflatoxin biosynthetic genes, aflD and aflR expression, and phenotypic aflatoxin B1 production by *Aspergillus flavus* under different environmental and nutritional regimes. Int. J. Food Microbiol..

[51] Harkai P., Szabó I., Cserháti M., Krifaton C., Risa A., Radó J., Balázs A., Berta K., Kriszt B. (2016). Biodegradation of aflatoxin-B1 and zearalenone by *Streptomyces* sp. collection. Int. Biodeterior. Biodegrad..

[52] Teniola O.D., Addo P.A., Brost I.M., Farber P., Jany K.-D., Alberts J.F., Van Zyl W.H., Steyn P.S. (2005). Degradation of aflatoxin B1 by cell-free extracts of *Rhodococcus erythropolis* and *Mycobacterium fluoranthenivorans* sp. nov. DSM44556T. Int. J. Food Microbiol..

[53] El Khoury R., Mathieu F., Atoui A., Kawtharani H., El Khoury A., Afif C., Maroun R.G., El Khoury A. (2017). Ability of soil isolated actinobacterial strains to prevent, bind and biodegrade ochratoxin A. Toxins.

[54] Manteca A., Alvarez R., Salazar N., Yagüe P., Sanchez J. (2008). Mycelium differentiation and antibiotic production in submerged cultures of *Streptomyces coelicolor*. Appl. Environ. Microbiol..

[55] Mannaa M., Kim K.D. (2017). Influence of temperature and water activity on deleterious fungi and mycotoxin production during grain storage. Mycobiology.

[56] Alberts J.F., Engelbrecht Y., Steyn P.S., Holzapfel W.H., Van Zyl W.H. (2006). Biological degradation of aflatoxin B1 by *Rhodococcus erythropolis* cultures. Int. J. Food Microbiol..

[57] Taylor M.C., Jackson C.J., Tattersall D.B., French N., Peat T.S., Newman J., Briggs L.J., Lapalikar G.V., Campbell P.M., Scott C. (2010). Identification and characterization of two families of F420H2-dependent reductases from Mycobacteria that catalyse aflatoxin degradation. Mol. Microbiol..

[58] Lapalikar G.V., Taylor M.C., Warden A.C., Scott C., Russell R.J., Oakeshott J.G. (2012). F 420H 2-dependent degradation of aflatoxin and other furanocoumarins is widespread throughout the *Actinomycetales*. PLoS ONE.

[59] Guan S., Ji C., Zhou T., Li J., Ma Q., Niu T. (2008). Aflatoxin B 1 degradation by *Stenotrophomonas maltophilia* and other microbes selected using coumarin medium. Int. J. Mol. Sci..

[60] Wang L., Wu J., Liu Z., Shi Y., Liu J., Xu X., Hao S., Mu P., Deng F., Deng Y. (2019). Aflatoxin B1 degradation and detoxification by *Escherichia coli* CG1061 isolated from chicken cecum. Front. Pharmacol..

[61] Zhu Y., Hassan Y.I., Lepp D., Shao S., Zhou T. (2017). Strategies and methodologies for developing microbial detoxification systems to mitigate mycotoxins. Toxins.

[62] Zhao L., Guan S., Gao X., Ma Q., Lei Y., Bai X., Ji C. (2011). Preparation, purification and characteristics of an aflatoxin degradation enzyme from *Myxococcus fulvus* ANSM068. J. Appl. Microbiol..

[63] Loi M., Fanelli F., Liuzzi V.C., Logrieco A.F., Mulè G. (2017). Mycotoxin biotransformation by native and commercial enzymes: Present and future perspectives. Toxins.

[64] Samuel M.S., Sivaramakrishna A., Mehta A. (2014). Degradation and detoxification of aflatoxin B1 by *Pseudomonas putida*. Int. Biodeterior. Biodegrad..

[65] Lee L.S., Dunn J.J., Delucca A.J., Ciegler A. (1981). Role of lactone ring of aflatoxin B1 in toxicity and mutagenicity. Experientia.

[66] Iram W., Anjum T., Iqbal M., Ghaffar A., Abbas M. (2015). Mass spectrometric identification and toxicity assessment of degraded products of aflatoxin B1 and B2 by *Corymbia citriodora* aqueous extracts. Sci. Rep..

[67] Iram W., Anjum T., Iqbal M., Ghaffar A., Abbas M., Khan A.M. (2016). Structural analysis and biological toxicity of aflatoxins B1 and B2 degradation products following detoxification by *Ocimum basilicum* and *Cassia fistula* aqueous extracts. Front. Microbiol..

[68] Iram W., Anjum T., Iqbal M., Ghaffar A., Abbas M. (2016). Structural elucidation and toxicity assessment of degraded products of aflatoxin B1 and B2 by aqueous extracts of *Trachyspermum ammi*. Front. Microbiol..

[69] Wall-Martínez H., Ramírez-Martínez A., Wesolek N., Brabet C., Durand N., Rodríguez-Jimenes G.C., García-Alvarado M.A., Salgado-Cervantes M.A., Robles-Olvera V.J., Roudot A. (2019). Risk assessment of exposure to mycotoxins (aflatoxins and fumonisins) through corn tortilla intake in Veracruz City (Mexico). Food Addit. Contam. Part A.

