# Supplementary material for: Aspergillus flavus Growth Inhibition and Aflatoxin B1 Decontamination by Streptomyces Isolates and Their Metabolites
Source: Toxins (Basel). 2021 May 8;13(5):340. doi: 10.3390/toxins13050340 (PMC8151643; doi:10.3390/toxins13050340)
Supplement: Supplementary file 1 [file toxins-13-00340-s001.zip › toxins-1191983 supp proof done.pdf]

Supplementary Materials: *Aspergillus flavus* Growth Inhibition and Aflatoxin B_1 Decontamination by *Streptomyces* Isolates and Their Metabolites

Ixchel Campos-Avelar, Alexandre Colas de la Noue, Noël Durand, Guillaume Cazals, Véronique Martinez, Caroline Strub, Angélique Fontana and Sabine Schorr-Galindo

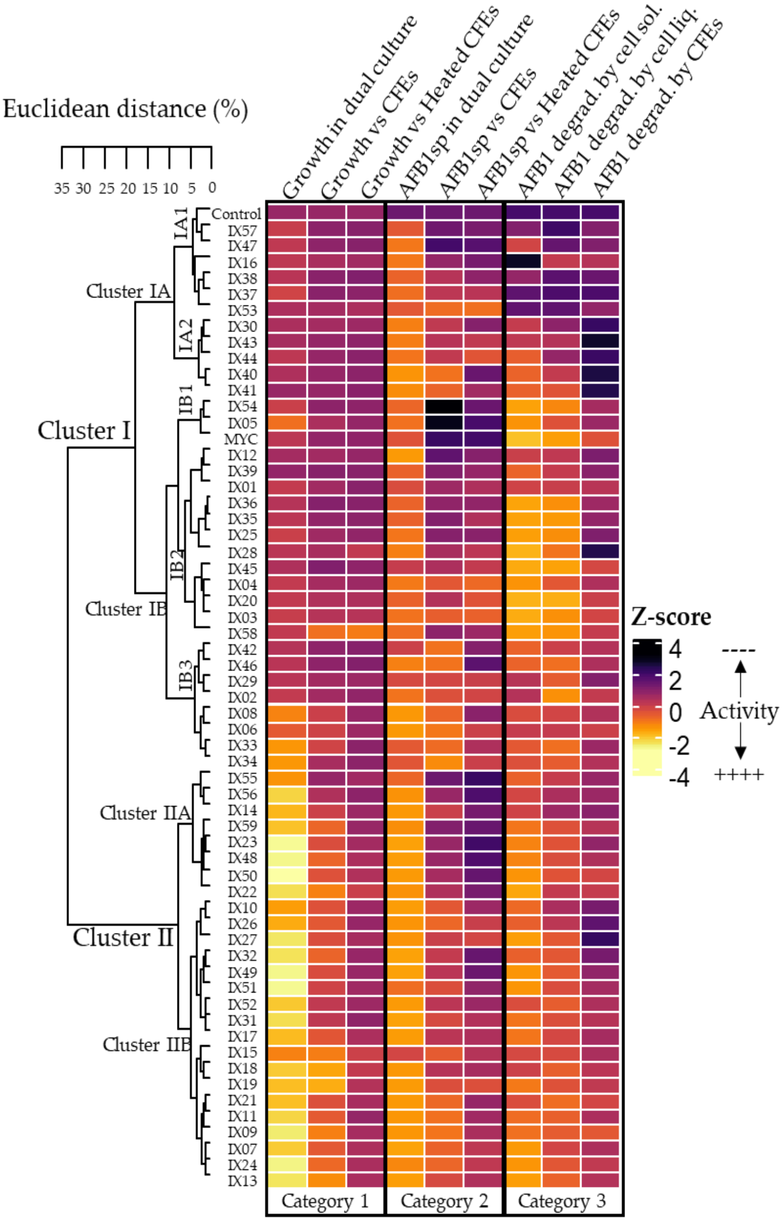

Figure S1. Heatmap of the effect of *Streptomyces* isolates and their CFEs on *Aspergillus flavus* growth (Category 1) and AFB_1 accumulation (Category 2), along with the bacteria's ability to degrade AFB_1 in solid and liquid medium and by their CFEs (Category 3). Results are given in a range of colours according to their Z-score, where purple to black represents a lack of activity or an increase compared to the control, whereas yellow represents the strongest activity.

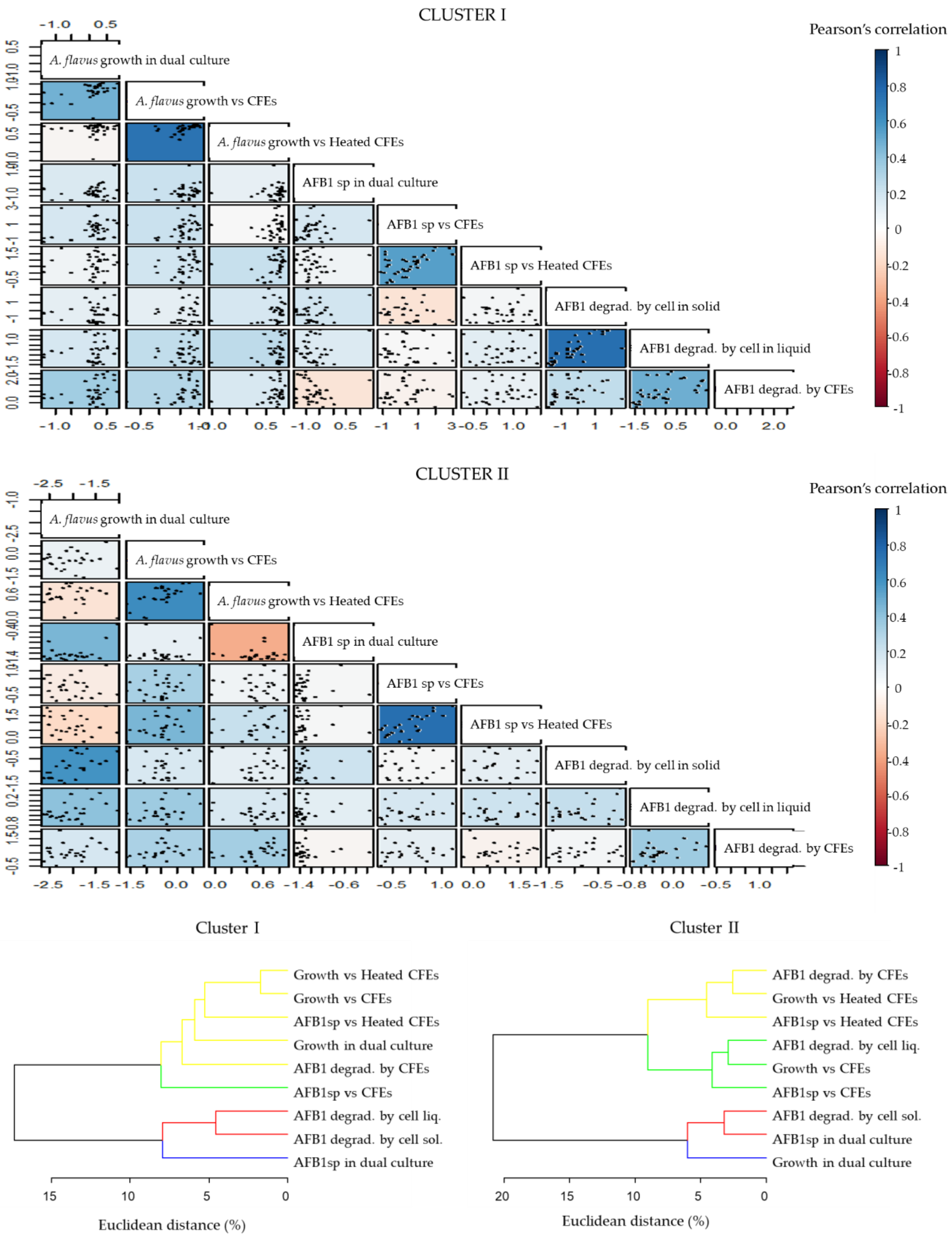

Figure S2. Pearson's correlation of the effects of *Streptomyces* isolates and their CFEs on *Aspergillus flavus* growth and AFB_1 accumulation, along with the degradation by bacteria in solid and liquid medium, and the degradation by their CFEs. The correlation scale goes from 1 in blue which indicates a strong positive correlation, to -1 in dark red that represents a strong negative correlation. The correlations were calculated for the isolates of each main cluster of the heatmap S1 (Cluster I and Cluster II). Values and significance are presented in supplementary Table S1. Dendrograms at the bottom show the relationship and proximity in % of Euclidean distance of each of the studied parameters for Clusters I and II. Different groups are indicated by different colours.

Table S1. Values of the Pearson correlation (*r*) between the measured parameters during the screening of the effect of *Streptomyces* isolates and Mycostop® and their CFEs on *A. flavus* growth and AFB1 accumulation.

	GDC	GCFE	GHCFE	AFBDC	AFBCFE	AFBHCFE	AFBDS	AFBDL	AFBDCFE	
**Cluster I**	1									GDC
	0.5 (.003)**	1								GCFE
	0 (.91)	0.7 (<.001)***	1							GHCFE
	0.2 (.37)	0.2 (.18)	0.1 (.64)	1						AFBDC
	0.2 (.32)	0.2 (.20)	0 (.99)	0.2 (.32)	1					AFBCFE
	0 (.79)	0.2 (.23)	0.2 (.17)	0 (.80)	0.6 (<.001)***	1				AFBHCFE
	0.1 (.57)	0.1 (.58)	0.2 (.31)	0.2 (.27)	-0.1 (.43)	0 (.91)	1			AFBDS
	0.2 (.32)	0.3 (.14)	0.3 (.13)	0.1 (.39)	0 (.98)	0.1 (.51)	0.8 (<.001)***	1		AFBDL
	0.4 (.04)*	0.3 (.09)	0.2 (.33)	-0.1 (.46)	0 (.85)	0.1 (.67)	0.3 (.14)	0.5 (.002)**	1	AFBDCFE
**Cluster II**	1									GDC
	0.1 (.75)	1								GCFE
	-0.1 (.51)	0.6 (<.001)***	1							GHCFE
	0.5 (.02)*	0.1 (.70)	-0.4 (.07)	1						AFBDC
	-0.1 (.71)	0.3 (.09)	0 (.83)	0 (.93)	1					AFBCFE
	-0.2 (.34)	0.5 (.02)*	0.2 (.25)	0 (.95)	0.8 (<.001)***	1				AFBHCFE
	0.6 (.0012)**	0.2 (.41)	0.1 (.63)	0.2 (.29)	0 (.96)	0.1 (.63)	1			AFBDS
	0.4 (0.03)*	0.4 (.07)	0.2 (.43)	0.1 (.60)	0.2 (.40)	0.2 (.28)	0.2 (.22)	1		AFBDL
	0.2 (.38)	0.3 (.10)	0.3 (.08)	0 (.96)	0.1 (.66)	0 (.88)	0 (.92)	0.4 (.07)	1	AFBDCFE

GDC = *A.* *flavus* growth in dual culture, GCFE = *A. flavus* growth vs Cell Free Extracts (CFEs), GHCFE = *A. flavus* growth vs Heated CFEs, AFBDC = AFB1 specific production (sp) in dual culture, AFBCFE = AFB1sp vs CFEs, AFBHCFE = AFB1sp vs Heated CFEs, AFBDS =AFB1 degradation by cells in solid medium, AFBDL = AFB1 degradation by cells in liquid medium, AFBDCFE = AFB1 degradation by CFEs. *P*-values are indicated in the parenthesis. * $P < 0.05$, ** $P < 0.01$, *** $P < 0.001$

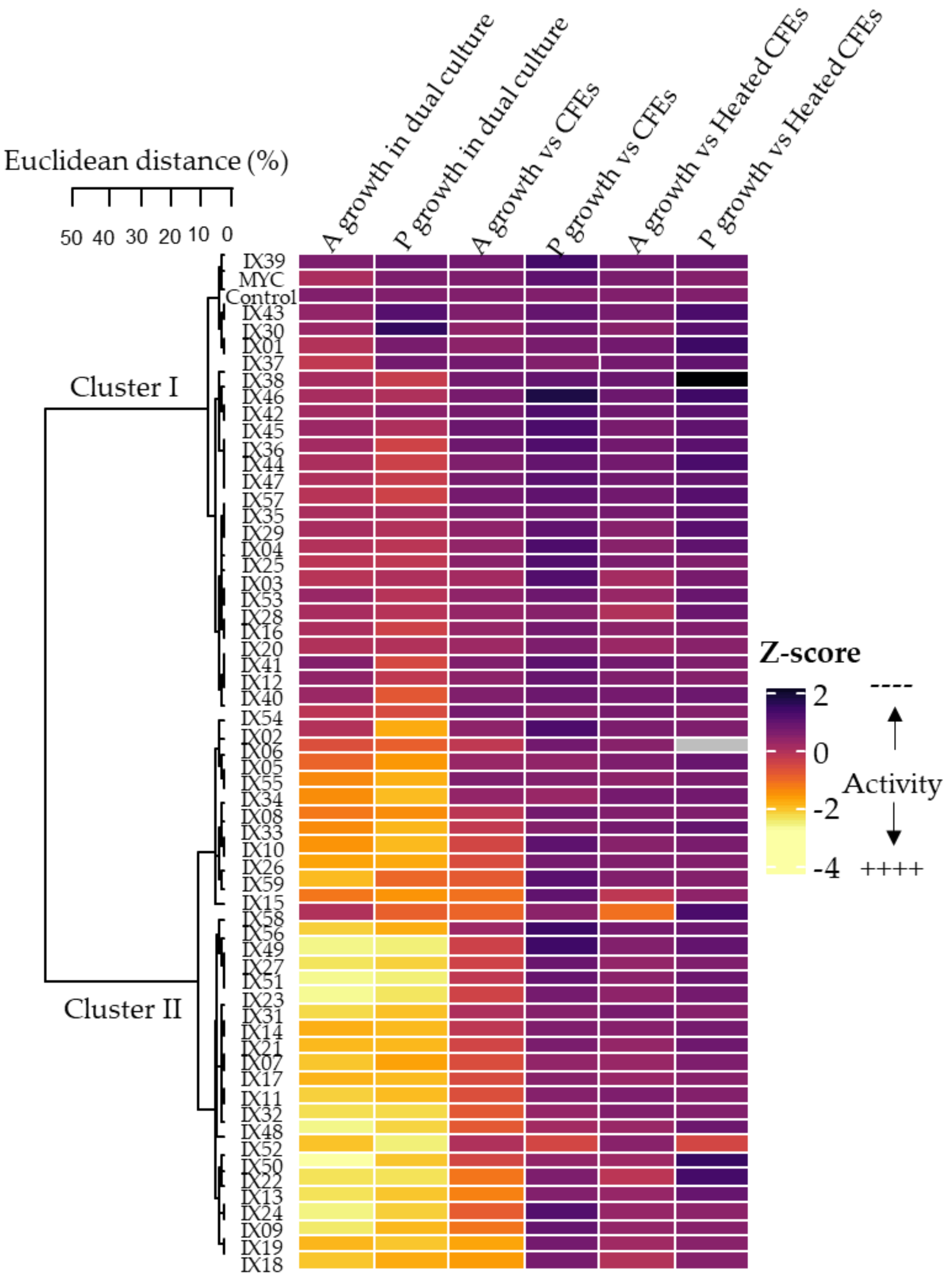

Figure S3. Heatmap of the effect of *Streptomyces* isolates and their CFEs on *Aspergillus flavus* (A) and *Penicillium verrucosum* (P) growth. Results are given in a range of colours according to their Z-score, where purple to black represents a lack of activity or an increase compared to the control, whereas yellow represents the strongest activity. Grey = missing value.

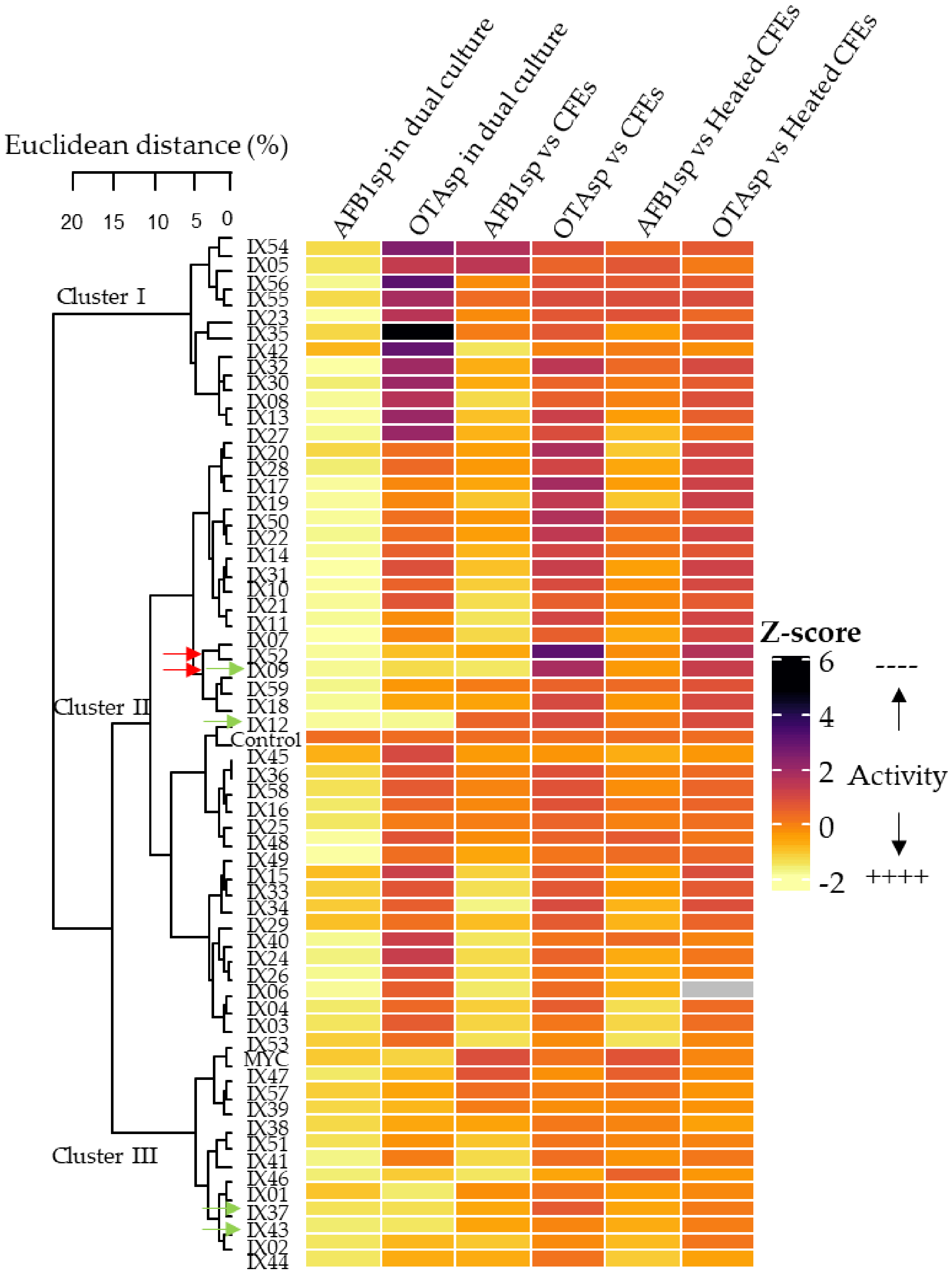

Figure S4. Heatmap of the effect of *Streptomyces* isolates and their CFEs on AFB_1 accumulation by *Aspergillus flavus* and on OTA accumulation by *Penicillium verrucosum*. Results are given in a range of colours according to their Z-score, where purple to black represents a lack of activity or an increase compared to the control, whereas yellow represents the strongest activity. Grey = missing value.

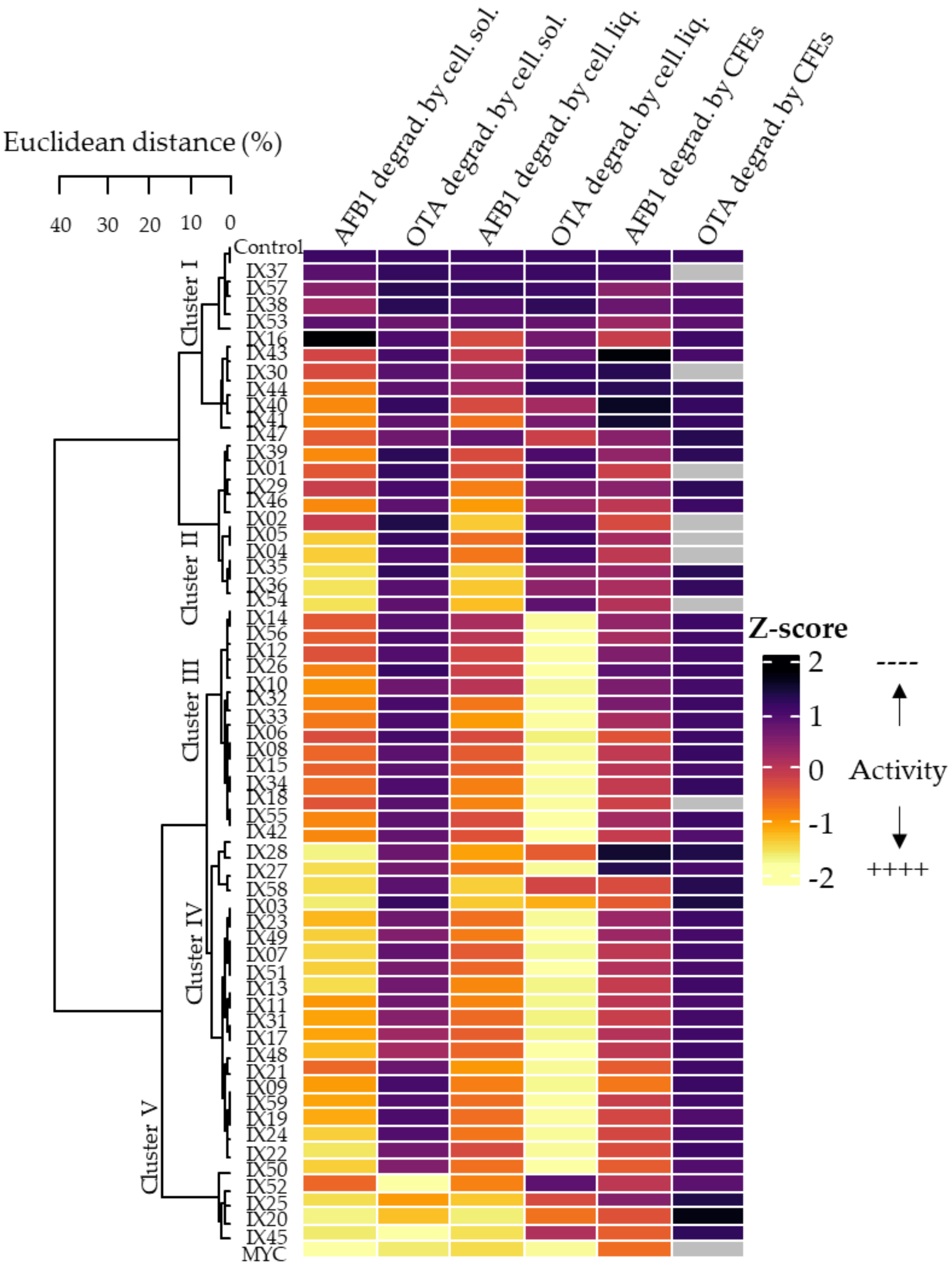

Figure S5. Heatmap of *Streptomyces* isolates and their CFEs ability to degrade AFB_1 and OTA. Results are given in a range of colours according to their Z-score, where purple to black represents a lack of activity or an increase compared to the control, whereas yellow represents the strongest activity. Grey = missing values.
